# Development of an interfering peptide M1-20 with potent anti-cancer effects by targeting FOXM1

**DOI:** 10.1038/s41419-023-06056-9

**Published:** 2023-08-19

**Authors:** Huitong Bu, Xianling Lan, Haojie Cheng, Chaozhu Pei, Min Ouyang, Yan Chen, Xiaoqin Huang, Li Yu, Yongjun Tan

**Affiliations:** State Key Laboratory of Chemo/Biosensing and Chemometrics, College of Biology, Hunan Engineering Research Center for Anticancer Targeted Protein Pharmaceuticals, Hunan University, Changsha, Hunan 410082 China

**Keywords:** Cancer therapy, Recombinant peptide therapy

## Abstract

Disrupting protein–protein interactions (PPIs) has emerged as a promising strategy for cancer drug development. Interfering peptides disrupting PPIs can be rationally designed based on the structures of natural sequences mediating these interactions. Transcription factor FOXM1 overexpresses in multiple cancers and is considered an effective target for cancer therapeutic drug development. Using a rational design approach, we have generated a peptide library from the FOXM1 C-terminal sequence and screened FOXM1-binding peptides. Combining FOXM1 binding and cell inhibitory results, we have obtained a FOXM1-targeting interfering peptide **M1-20** that is optimized from the natural parent peptide to the D-retro-inverso peptide. With improved stability characteristics, **M1-20** inhibits proliferation and migration, and induces apoptosis of cancer cells. Mechanistically, **M1-20** inhibits FOXM1 transcriptional activities by disrupting its interaction between the MuvB complex and the transcriptional co-activator CBP. These are consistent with the results that **M1-20** suppresses cancer progression and metastasis without noticeable toxic and side effects in wild-type mice. These findings reveal that **M1-20** has the potential to be developed as an anti-cancer drug candidate targeting FOXM1.

## Introduction

Transcription factors are associated with the initiation and progression of cancers, and are recognized as potential targets for developing novel cancer therapeutics [1]. Modulating the expression and stability of transcription factors, or interfering with their binding to DNA sequences, can affect their activity [2]. However, disrupting protein–protein interactions (PPIs) is a promising strategy for cancer drug development [3], particularly through the use of interfering peptides that bind to the large, flat contact surfaces mediating PPIs. In practice, interfering peptides that abolish the functions of transcription factors can be predicted by so-called rational design strategies, wherein peptides are produced based on the natural amino acid sequences of PPI surfaces [4]. So far, several interfering peptides targeting transcription factors have been validated for cancer treatment in preclinical studies (e.g., TCF4 N-terminus-derived peptide targeting β-catenin:TCF interaction [5], p73 DBD-derived peptide targeting p53:p73 interaction [6], and GAPDH-derived peptide targeting p53:GAPDH interaction [7]) or in clinical trials (e.g., NSC745104 targeting p53:HDM2 interaction [8] and Omo-103 targeting C-Myc:Max interaction [9]). All these interfering peptides have been rationally designed based on the structures of natural sequences that mediate the PPIs of transcription factors.

FOXM1 is a member of the forkhead box (FOX) transcription factor family [10], and expresses in all developmental embryo tissues but only in adult tissues with a high proliferation index [11]. In multiple cancers, FOXM1 is upregulated and its levels in clinical cancer samples can predict disease diagnosis and prognosis [12]. FOXM1 stimulates the cell cycle, promoting malignant proliferation through the transcriptional activation of genes involved in G1/S and G2/M transitions [13]. It is also involved in cancer cell metastasis [14], and maintaining the characteristics of cancer stem cells [15]. Additionally, FOXM1 stimulates the expression of DNA damage repair-related genes in cancer cells [16], while inhibition of FOXM1 can enhance the sensitivity of cancers to chemical therapeutics [17]. As a survival protein, FOXM1 specifically protects cancer cells from apoptosis by upregulating antiapoptotic genes [18]. Given its multiple roles in promoting cancer progression, FOXM1 is considered a potential target for cancer therapy development [19]. Several small molecule compounds, including Thiostrepton [20], RCM [21], antibiotic Siomycin A [22], and FDI-6 [23], can impair the growth of cancer cells by disrupting FOXM1 transcriptional activities. A FOXM1 DBD-specific single-strand DNA aptamer has also been selected to inhibit FOXM1 transcriptional functions in cancer cells [24]. Besides, a recombinant protein M1-138 has been generated to target FOXM1 in cancer cells by fusing the FOXM1 N-terminal domain (1-138aa) with a cell-penetrating peptide [25]. Currently, there are no commercially available anti-cancer therapeutics specifically targeting FOXM1.

Two mechanisms by which FOXM1 activates transcription have been established. First, FOXM1 directly binds to its conserved DNA motif of RYAAAYA (FKH motif) in downstream gene promoters, such as CDC25B, through its DNA-binding domain (DBD, 232-332aa) to activate their transcription [26]. Second, FOXM1 can stimulate gene expression through an indirect DNA binding mechanism mediated by the MuvB complex [27]. This complex, composed of multiple subunits including LIN9, binds to the CHR (Cell cycle genes Homology Region) motif (TTTGAA or TTTAAA) [28] in target gene promoters and controls precisely timed transcription of the cell cycle. LIN9 in the MuvB complex interacts with the FOXM1 N-terminal domain and recruit FOXM1 to the promoters of certain cell cycle genes, like PLK1, enabling its target gene transcription without direct DNA binding [29]. The transcriptional activities of FOXM1 also rely on its C-terminal domain that interacts with the transcription co-activator CBP [30], which is essential for inducing gene transcription through acetylating histone [31] and initiating RNA Pol II basal complex [32]. In addition, the FOXM1 N-terminal domain can interact with other cancer-related transcription factors, such as β-catenin or Smad3, facilitating their nuclear import process to fully activate the classical WNT or TGF-β signaling pathway [33, 34].

In this study, we intend to develop interfering peptides targeting FOXM1 for cancer treatment. The FOXM1 C-terminal domain is an ideal target for screening interfering peptides as it interacts with the N-terminal domain, which mediates PPIs with multiple partner proteins [35]. These peptides may also have the potential to disrupt FOXM1 transcriptional activities by competing with FOXM1:CBP interaction. Based on the rational design strategy, we have developed an interfering peptide (**M1-20**) that potently binds to FOXM1 and inhibits its transcriptional activities by disrupting interactions between the FOXM1 N-terminal domain and the MuvB complex or between FOXM1 and CBP. **M1-20** inhibits cancer cell proliferation and migration and induces apoptosis of cancer cells in vitro and in vivo, without obvious toxic and side effects, indicating its potential for developing anti-cancer drugs.

## Materials and methods

### Cell culture

HEK293T, MDA-MB-231, MCF7, ZR-75-30, MCF10A, Hela, U2OS, A549, and 4T1 cells were obtained from ATCC (Manassas, USA). Details were described in the supplemental materials and methods.

### Luciferase reporter assay

The luciferase enzyme activities were measured with the Dual-Luciferase Reporter Assay System (Promega, USA) following the manufacturer’s instructions. The detailed protocols were described in the supplemental materials and methods

### Solid-phase synthesis of peptides

Peptides were generated in a solid phase synthesizer (CS Bio) with 2-Chlorotrityl Chloride resin as solid phase support following the instrument operation program. Details were described in the supplemental materials and methods.

### Structural modeling with Rosetta FlexPepDock

The N-terminal structure of FOXM1 (PDB code 6OSW) was obtained from the Protein Data Bank database (PDB, https://www.rcsb.org). The structural model of peptide and FOXM1 was analyzed by Rosetta FlexPepDock [36], InterfaceAnalyzer [37], and Flex ddG [38]. Details were described in the supplemental materials and methods.

### Microscale thermophoresis assay

Measurements were performed by Monolith NT.115 instrument (NanoTemper). The data were analyzed by MO. Affinity Analysis v2.3 NT software (NanoTemper) to determine interaction parameters. Details were described in the supplemental materials and methods.

### Pull-down and co-immunoprecipitation (Co-IP) assays

Pull-down experiments were performed using Ni-Sepharose^TM^ 6 Fast Flow (GE Healthcare, USA) and Streptavidin Agarose Resin (GE Healthcare, USA). Co-IP experiment was performed using Flag magnetic beads (Bimake, USA). Details were described in the supplemental materials and methods.

### Protein extraction and western blotting

The cells were lysed on ice with IP lysis buffer and tumor tissue samples were homogenized, grinded, and lysed on ice with RIPA buffer. The protein concentration of lysates was quantified using a BCA Protein Assay kit (Thermo Fisher Scientific, USA). Protein lysates were separated by SDS-PAGE gel electrophoresis and transferred to 0.22 µm PVDF membranes (Merck Millipore, USA), followed by Western blotting with certain antibodies. Details were described in the supplemental materials and methods. Antibody information is available in Supplemental information Table S1. Uncropped immunoblot gels are shown in Original WB data.

### RNA isolation and real-time quantitative PCR (RT-PCR)

TRIzol reagent (Invitrogen, USA) was used to extract the total RNA of cells according to the manufacturer’s instructions. cDNA was synthesized from total RNA (2 μg) by reverse transcription (Thermo Fisher Scientific, USA) according to the instructions provided by the manufacturer. RT-PCR was performed using SYBR QPCR Master Mix (Vazyme) with certain sense (S) and antisense (AS) primers, and realplex2 qPCR system (Eppendorf, Germany). The information on RT-PCR primer pairs was presented in Supplemental information Table S2.

### RNA sequencing

MDA-MB-231 cells were treated with **M1-20** (10 µm) or **M1-20mut** (10 µm) for 24 h and collected into TRIzol reagent (Invitrogen, USA) and sent to Majorbio (Shanghai, China) for RNA extraction and sequencing. Details were described in the supplemental materials and methods.

### The anti-cancer effects of M1-20 in vivo

Healthy ICR/JCL mice (6-week-old), BALB/c mice (female, 4-week-old), and BALB/c nude mice (female, 4-week-old) were purchased from Hunan Slac Laboratory Animal Company (Changsha, China). The detailed procedure was described in Supplementary Materials and Methods.

### Ethics approval

All animal care and experiments were performed by the guidelines approved by the Laboratory Animal Center of Hunan, China (Protocol No. SYXK [Xiang] 2018-0006).

### Statistical analysis

Data analysis and visualization were performed using Microsoft Excel and GraphPad Prism 9 (GraphPad Software Inc.). The analysis was tested by unpaired t-test between two groups, and by one-way ANOVA or two-way ANOVA with multiple comparisons in multiple groups. *P* < 0.05 was considered statistically significant.

## Results

### FOXM1_689-748_ inhibited the transcriptional activities of FOXM1

**Fig. 1:**
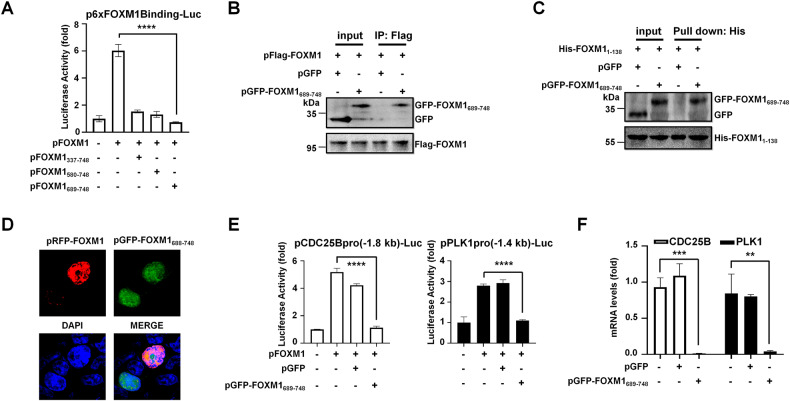
FOXM1_689-748_ inhibited the transcriptional activities of FOXM1. **A** The reporter plasmid, containing 6×FOXM1 binding sequences in its promoter (p6×FOXM1Binding-Luc, 1 µg) were transfected with pFOXM1 (0.3 µg) and different length of FOXM1 C-terminus (pFOXM1_337-748_, pFOXM1_580-748_, or pFOXM1_689-748_, 0.3 µg) into HEK293T cells, plus pRL-CMV plasmid (20 ng/well) as a loading control. After 48 h, cell lysates were collected and prepared for measurement of dual Luciferase activity. *n* = 3 for each group, ****P < 0.0001, two-tailed unpaired Student’s t-test. **B** HEK293T cells were transfected with pFlag-FOXM1 and pGFP or pGFP-FOXM1_689-748_ and 48 h later cell lysates were harvested. The lysates (500 μg) were incubated with Flag magnetic beads to immunoprecipitate Flag-FOXM1/protein complexes which were analyzed by Western Blotting with certain antibodies. 10% of cell lysates (50 μg) were used as input controls. **C** HEK293T cells were transfected with pGFP-FOXM1_689-748_ or pGFP for 48 h. Purified protein His-FOXM1_1-138_ added to His-tag resin were incubated with cell lysates (500 µg) overnight at 4 °C. Pull-downs were analyzed by Western Blotting with Flag-tag and His-tag antibodies. 10% of cell lysates (50 μg) were used as input controls. **D** HEK293T cells were transfected with pRFP-FOXM1 and pGFP-FOXM1_689-748_ and 48 h later cells were fixed with 4% paraformaldehyde. The localization of GFP-FOXM1 and GFP-FOXM1_689-748_ was imaged with the fluorescence confocal microscope (Olympus FluoView FV1200). The DAPI signal indicated the location of the cell nucleus. **E** Reporter plasmids (1 µg) containing the −1.8 kb CDC25B promoter-luciferase reporter (left) or the −1.4 kb PLK1 promoter-luciferase reporter (right) were transfected with pFOXM1 (0.3 µg) and pGFP (0.3 µg) or pGFP-FOXM1_689-748_ (0.3 µg) into HEK293T cells, plus pRL-CMV plasmid (20 ng/well) as a loading control. Cell lysates were prepared after 48 h and respectively used for the measurement of dual Luciferase activity. *n* = 3 for each group, ****P < 0.0001, two-tailed unpaired Student’s t-test. **F** HEK293T cells were transfected with pGFP or pGFP-FOXM1_689-748_ and 48 h later cells were collected for the preparation of total RNA. The mRNA levels of CDC25B and PLK1 were examined by RT-PCR. Relative mRNA levels were normalized to GAPDH. *n* = 3 for each group, **P < 0.01, ***P < 0.001, two-tailed unpaired Student’s t-test.

Based on the FOXM1 C-terminal domain mediated by multiple PPIs, we constructed a series of expression vectors with the different length of FOXM1 C-terminus (pFOXM1_337-748_, pFOXM1_580-748_, and pFOXM1_689-748_). We found that the overexpression of the each FOXM1 fragment inhibited the transactivation activity of FOXM1 to its luciferase reporter vector (p6×FOXM1Binding-Luc), in which FOXM1_689-748_ showed the best inhibitory effect (Fig. 1A). FOXM1_1-688_, in which the 689-748aa sequence was deleted from FOXM1, also lost transcriptional activity compared with full-length FOXM1 (Supplemental Fig. 1A). Co-immunoprecipitation (Co-IP) experiments showed that GFP-tagged FOXM1_689-748_ interacted with exogenously expressed Flag-FOXM1 in cells (Fig. 1B) and in particular with FOXM1 N-terminus (His-FOXM1_1-138_) (Fig. 1C). Recombinant protein GST-FOXM1_689-748_ could pull down FOXM1 proteins from cell extracts (Supplemental Fig. 1B). We transfected HEK293T cells with expression vectors pGFP-FOXM1_689-748_ and pRFP-FOXM1, and observed that they co-localized in the nucleus (Fig. 1D). We performed co-transfection experiments and Luciferase assays to show that FOXM1 activated FKH-containing −1.8 kb CDC25B promoter [26] or CHR-containing −1.4 kb PLK1 promoter [28], while the expression of FOXM1_689-748_ abolished the stimulation of FOXM1 on both promoters (Fig. 1E). The mRNA levels of CDC25B and PLK1 were also decreased in the FOXM1_689-748_ expressed cells (Fig. 1F). Thus, the sequence 689-749aa of FOXM1 provided a potential natural amino acid sequence for screening FOXM1 interfering peptides.

### The screening of the FOXM1-interacting peptide M1-20

**Fig. 2:**
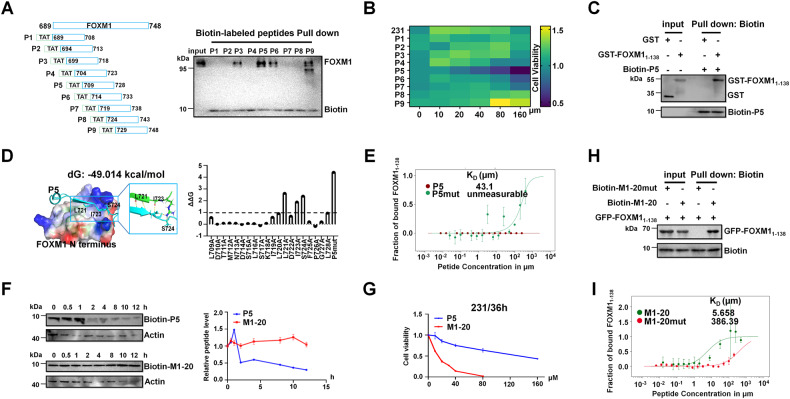
The screening of the FOXM1-interacting peptide M1-20. **A** The diagram of peptide array (**P1**-**P9**) in which peptides (20-mer) covering FOXM1_689-748_ with a shifting window of five residues were conjugated to the cell-penetrating peptide TAT and produced by solid-phase peptide synthesis. The **P1**-**P9** peptides were biotin-labeled respectively and added in MDA-MB-231 cell lysates (500 μg) which were incubated with Streptavidin Agarose Resin to pull down biotin-peptide/protein complexes. Biotin and FOXM1 proteins in samples were detected by Western Blotting with certain antibodies. 10% of cell lysates (50 μg) were used as input controls. **B** MDA-MB-231 cells (4 × 10^3^ cells/well) were seeded in 96-well plates for 12 h and treated with a defined concentration gradient of the **P1**-**P9** peptides (0, 10, 20, 40, 80, 160 µM). 36 h later, CCK-8 solution (10%) was added to each well and incubated for another 2 h. The absorbance at 450 nm and the relative cell viability in each well was calculated. The percentage of cell activity versus the concentration of peptides was plotted by heatmap. *n* = 3 for each group. **C** Recombinant protein GST-FOXM1_1-138_ (50 μg) was added to biotin-labeled **P5** (10 μg) which was incubated with Streptavidin Agarose Resin to pull down biotin-peptide/protein complexes. GST protein was used as a control. GST-tag and Biotin-tag were detected by Western Blotting with certain antibodies. 10% of recombinant protein was used as input controls. **D** The model of **P5** binding to the N-terminus of FOXM1 (PDB ID 6OSW) was built by Rosetta FlexPepDock. Left, the interface of peptide-protein interaction, the peptide was shown in cyan, and the N-terminus of FOXM1 was shown as electrostatic potential in Protein. Right, hydrogen bonds were formed by core residues at the interface of peptide and protein interaction. The curve was shown the free energy change (ΔΔG) by single point mutation with alanine scanning using Rosetta Flex ddG (ΔΔG ≥ 1, binding energy decreases; 1 > ΔΔG > −1, binding energy no changes; ΔΔG ≤ −1, binding energy increases). **E** The curve represented the quantification of binding affinity between GFP-labeled FOXM1_1-138_ and **P5** or **P5mut** by Microscale Thermophoresis (MST, Monolith NT.115, NanoTemper). Data points indicated the fraction of FOXM1_1-138_-bound peptide (ΔNormal/Amplitude) at different concentrations, and curves indicated the calculated fits. Error bars represent the SE of three independent measurements. Mean values of binding affinity were shown on the panel. **F** The biotin signals indicated the peptide stability of **P5** and **M1-20** in HEK293T cell lysates analyzed by Western Blotting. The levels of peptide were quantified by Image J software and graphed with GraphPad Prism 9 (*n* = 3). **G** MDA-MB-231 cells were seeded in 96-well plates for 12 h and treated with different concentrations of **P5** or **M1-20**. 36 h later CCK-8 solution (10%) was added to each well and incubated for another 2 h. The absorbance at 450 nm and the relative cell viability in each well was calculated and plotted by GraphPad Prism 9. *n* = 3 for each group. **H** Recombinant protein His-FOXM1_1-138_ (50 μg) added to biotin-labeled **M1-20** (10 μg) or **M1-20mut** (10 μg) was incubated with Streptavidin Agarose Resin to pull down biotin-peptide/protein complexes. GFP-tag and Biotin-tag in samples were detected by Western Blotting with certain antibodies. 10% of the recombinant protein/peptide complex was used as input controls. **I** Quantification of binding affinity between GFP-labeled FOXM1_1-138_ and **M1-20** or **M1-20mut** by Microscale Thermophoresis. Mean values of binding affinity were shown on the panel. Experiments were repeated three times with similar results.

We created a peptide library containing **P1** to **P9** peptides (20-mers, conjugated with TAT cell-penetrating sequence) with a moving window of five residues covering the sequence 689-748aa of FOXM1, for screening FOXM1-interacting peptides (Fig. 2A). Western blotting showed that **P3,** **P5,** **P6**, and **P9** interacted with endogenous FOXM1 with variable affinities (Fig. 2A). Further investigation showed that only **P5** (the sequence 709-728aa of FOXM1) (Supplemental Fig. 2A) significantly inhibited MDA-MB-231 breast cancer cells in a dose-dependent manner (Fig. 2B), providing a reason to focus on this peptide for further studies. Biotin-labeled **P5** was found to bind to recombinant GST-FOXM1_1-138_ protein but not GST control protein, confirming its ability to interact with FOXM1_1-138_ (Fig. 2C). Rosetta FlexPepDock [36] was used to simulate a structure model for **P5** binding to FOXM1_1-138_ and Rosetta InterfaceAnalyzer [37] was performed to calculate the docking free energy (the value of dG around −49 kcal/mol) for the interface between **P5** and FOXM1_1-138_ (Fig. 2D). Single point mutation with alanine scanning by Rosetta Flex ddG [38] revealed that the three residues in **P5** (L721, I723, and S724 of FOXM1) were predicted as the core sites for mediating **P5** interaction with FOXM1_1-138_ (Fig. 2D). Mutating the three residues in **P5** to obtain **P5mut** (L721A, I723A, S724A) (Supplemental Fig. 2B) led to loss of FOXM1_1-138_ binding ability (the value of free energy change ΔΔG > 1 kcal/mol) (Fig. 2D). Microscale Thermophoresis was performed to measure the binding affinity of **P5** to FOXM1_1-138_ with K_D_ = 43.1 μM, while the binding of **P5mut** to FOXM1_1-138_ was unmeasurable (Fig. 2E). Next, we adopted the D-retro-inverso (DRI) strategy, in which DRI peptides were composed of D-amino acid assembled in the reverse order of natural parent peptides [39], to optimize the stability of **P5**. We synthesized the DRI form of **P5** to obtain **M1-20** (Supplemental Fig. 3A), which maintained a similar secondary structure as the parent **P5** measured by Circular Dichroism (Supplemental Fig. 4). Compared with **P5, M1-20** showed much better resistance to degradation in HEK293T cell lysates (Fig. 2F), and a stronger inhibitory ability to MDA-MB-231 cells (Fig. 2G). Pull-down results showed that FOXM1_1-138_ was bound by **M1-20** but not **M1-20mut** (Fig. 2H, Supplemental Fig. 3B). Microscale Thermophoresis further confirmed the binding ability of **M1-20** to FOXM1_1-138_ with K_D_ = 5.658 µM, much stronger than that of **M1-20mut** (K_D_ = 386.39 µM) or parent **P5** (K_D_ = 43.1 µM) (Fig. 2I and see above).

### M1-20 suppressed multiple types of cancer cells and affected multiple cancer cell phenotypes

**Fig. 3:**
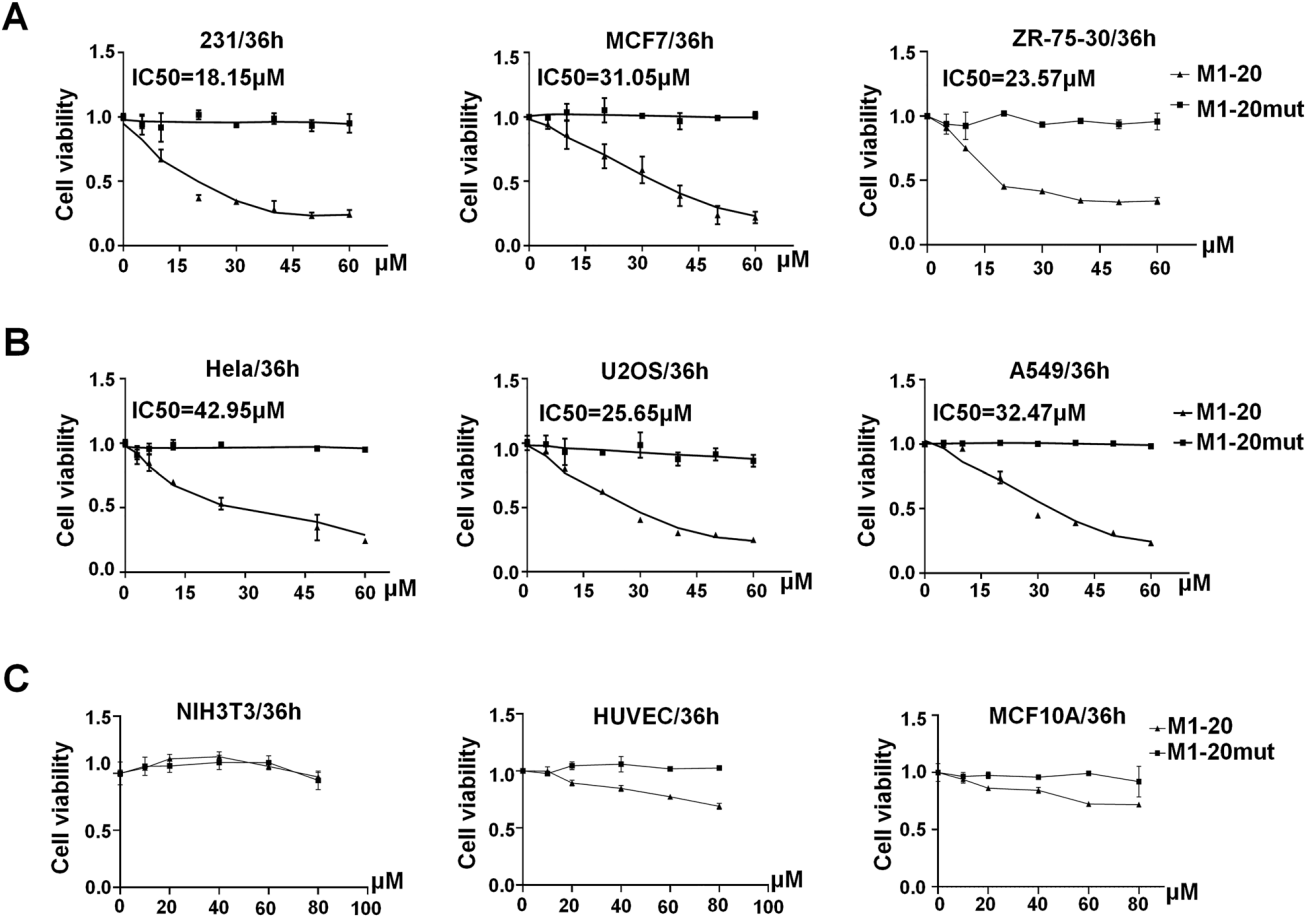
M1-20 suppressed the growth of cancer cells. **A** Cell viability of breast cancer cells (MDA-MB-231, MCF7, ZR-75-30 cells) after incubation with different **M1-20** concentrations for 36 h. **B** Cell viability of various cancer cells (Hela, U2OS, A549 cells) after treatment of **M1-20** for 36 h. **C** Cell viability of normal cells (NIH3T3, HUVEC, MCF10A cells) after treatment of **M1-20** for 36 h. Cells (4 × 10^3^ cells/well) were seeded in 96-well plates for 12 h and treated with a defined concentration gradient of **M1-20** or **M1-20mut** (5, 10, 20, 30, 40, 50, 60, 80 µM). 36 h later CCK-8 solution (10%) was added to each well and incubated for another 2 h. The absorbance at 450 nm and the relative cell viability in each well was calculated. The percentage of cell activity versus the concentration of **M1-20** was plotted (*n* = 3 for each group). The IC50 value of **M1-20** for each cell line was calculated with GraphPad Prism 9.

We selected multiple types of cancer cell lines and treated with **M1-20** by a defined concentration gradient (0, 10, 20, 40, 60, 80 µM) for 36 h. Compared with **M1-20mut,** **M1-20** significantly inhibited all of cancer cells in a dose-dependent manner (Fig. 3). The values of IC50 of **M1-20** were calculated for each cell line (18.15 µM for MBA-MD-231, 31.05 µM for MCF-7, 23.57 µM for ZR-75-30, 42.95 µM for Hela, 25.65 µM for U2OS, 32.47 µM for A549), showing variable sensitivities for different cancer cells responding to the **M1-20** treatment. Interestingly, we also noticed that **M1-20** resulted in no or mild inhibition of normal cell lines at the tested dosages (Fig. 3C), implicating relative safety for **M1-20** use in vivo. In the meantime, we demonstrated that cells with higher FOXM1 protein levels exhibited greater sensitivity to **M1-20** treatment while displaying lower IC50 values of **M1-20** (Supplemental Fig. 5A). In addition, the overexpression of FOXM1 in MCF7 or Hela cells with relatively low endogenous levels of FOXM1 resulted in heightened sensitivity towards **M1-20** (Supplemental Fig. 5B, C), supporting that **M1-20**’s anti-cancer efficacy correlated with the FOXM1 levels in cancer cells.

**Fig. 4:**
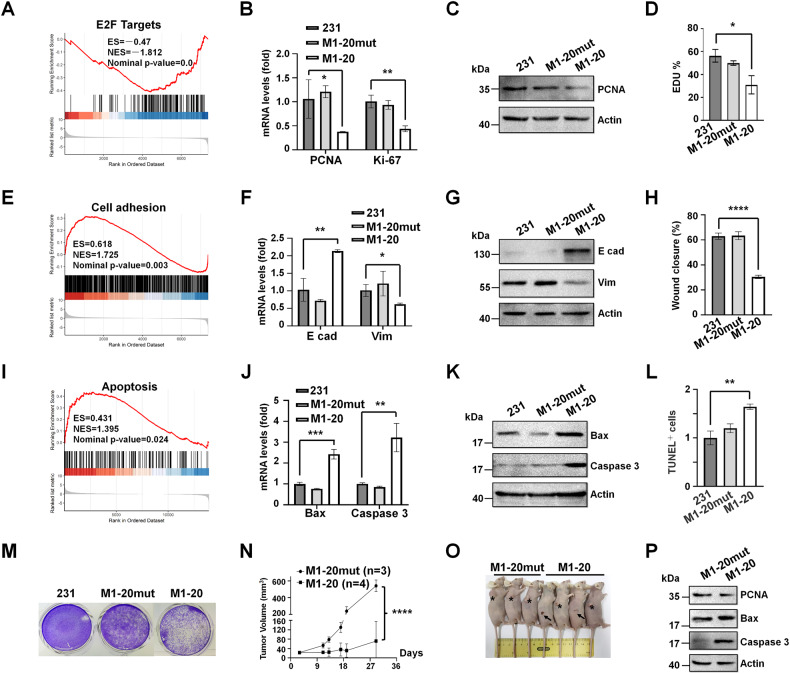
M1-20 inhibited the proliferation and migration of cancer cells, and induced the apoptosis of cancer cells. **A** MDA-MB-231 cells were treated with **M1-20** (10 µM) or **M1-20mut** (10 µM) and 24 h later the cells were collected in Trizol to extract the total RNA for RNA sequencing. The gene set of cell cycle (E2F targets) was significantly downregulated by **M1-20** in gene set enrichment analysis (GSEA) using differentially expressed genes (DEGs). **B**, **C** MDA-MB-231 cells were treated with **M1-20** (10 µM) or **M1-20mut** (10 µM) and 24 h later the cells were collected for the preparation of total RNA (**B**) and total proteins (**C**). The mRNA levels of PCNA and Ki-67 were examined by RT-RCR. Relative mRNA levels were normalized to GAPDH. *n* = 3 for each group, *P ≤ 0.05, **P < 0.01, ***P < 0.001, two-tailed unpaired Student’s t-test. Protein levels were measured by Western blotting with certain antibodies. **D** MDA-MB-231 cells were treated with **M1-20** (10 µm) for the detection of cell proliferation with EdU. Mean fluorescence intensity of EdU was measured by Image J (*n* = 3, *p < 0.05). **E** The gene set of cell adhesion was significantly upregulated by **M1-20** in GSEA analysis. **F**, **G** MDA-MB-231 cells were treated as (**B**), (**C**). The mRNA levels (**F**) and protein levels (**G**) of E-cadherin (E cad) and Vimentin (Vim) were respectively examined by RT-PCR and Western blotting. **H** MDA-MB-231 cells were treated with **M1-20** (10 µm) for the detection of cell migration by wound-healing assay. The cell migration area was calculated by Image J (*n* = 3, ****P ≤ 0.0001). **I** The gene set of apoptosis was significantly upregulated by **M1-20** in GSEA analysis. **J**, **K** MDA-MB-231 cells were treated as (**B**), (**C**). The mRNA levels (**J**) of Bax and Caspase 3 were examined by RT-PCR. Protein levels (**K**) were measured by Western blotting with certain antibodies. **L** MDA-MB-231 cells were treated with **M1-20** (10 µm) and detected apoptosis by TUNEL. TUNEL-positive cell content was quantified between the groups (*n* = 3, **P ≤ 0.01). **M** MDA-MB-231 cells (400 cells/well) were seeded in 6-well plates and treated with **M1-20** (10 µM) or **M1-20mut** (10 µM) for 14 days. The cells were fixed with 4% paraformaldehyde, stained with 0.1% crystal violet, and imaged. **N** The nude mice (female, 4–6 weeks old) were subcutaneously (S.C.) injected with MDA-MB-231 cells (1 × 10^7^ cells/mouse). When the tumor volume reached about 20 mm^3^, the mice were randomized into two groups (at least three mice per group) and orthotopically injected with **M1-20mut** (4 mg/kg) or **M1-20** (4 mg/kg). The volume of tumors was measured twice weekly using electronic and growth curves were obtained on Day 29. Tumor volume (*V*) was calculated by: *V* = length × diameter^2^ × 1/2. Significant differences were shown, 2-way ANOVA (Sidak’s multiple-comparisons test): ****P ≤ 0.0001. **O** At the end of the experiment, mice were imaged. Tumor localization in mice was marked with asterisks and tumor disappearance in two mice of the **M1-20** group was indicated by arrows. **P** The protein levels of PCNA, Bax, and Caspase 3 in collected tumor tissues were measured by western blotting.

Next, we focused on MDA-MB-231 cells for analyzing the effects of **M1-20** on the cellular processes of cancer cells. From the RNA-sequencing data of **M1-20** or **M1-20mut**-treated MDA-MB-231 cells (10 µM for 24 h), we noticed that multiple signaling pathways were significantly changed based on the gene set enrichment analysis (GSEA) using differentially expressed genes (DEGs) (Supplemental Fig. 6A). The gene sets of cell cycle (E2F targets) were suppressed in **M1-20**-treated cells (Fig. 4A), predicting that the proliferation of the cells was affected by **M1-20**. We found that the mRNA levels of Ki-67 and the mRNA and protein of PCNA were downregulated by **M1-20** (10 μM for 24 h) in MDA-MB-231 cells (Fig. 4B, C), correlated with the decreased proliferation of the cells in EdU staining experiments (Fig. 4D, Supplemental Fig. 6B). This finding was further supported by flow cytometry analysis, in which M1-20 reduced S-phase fraction and increased G1-phase fraction in the cells (Supplemental Fig. 6C), correlated with the decreased levels of CyclinD1, CyclinB1, and PLK1 (Supplemental Fig. 6D). The gene set of cell adhesion was activated by **M1-20** (Fig. 4E). The mRNA levels and protein levels of the epithelial marker E-cadherin enhancing cell adhesion were elevated and the levels of the mesenchymal marker Vimentin abolishing cell adhesion were declined by **M1-20** (Fig. 4F, G), correlated with the decreased migration of the cells in Wound Healing assays (Fig. 4H, Supplemental Fig. 5E). In addition, the gene set of apoptosis was activated by **M1-20** (Fig. 4I). The mRNA levels and protein levels of Bax and Caspase 3 were upregulated by **M1-20** (10 μM for 24 h) in MDA-MB-231 cells (Fig. 4J, K). Consequently, elevated levels of sub-G1 apoptotic bodies (Supplemental Fig. 6C), TUNEL staining (Fig. 4L, Supplemental Fig. 6F), and Annexin-positive apoptotic cells (Supplemental Fig. 6G) were observed in MDA-MB-231 cells treated with **M1-20**. **M1-20** inhibited the colony formation of MDA-MB-231 cells (Fig. 4M). To further test whether **M1-20** suppressed the progression of cancers in vivo, we made mouse cancer-engrafted models by subcutaneously implanting MDA-MB-231 cells (1 × 10^7^ cells/mouse) into BALB/c nude mice. We found that treatment with **M1-20** resulted in dramatic cancer suppression (Fig. 4N, O), consistent with the changed levels of the marker proteins specific for proliferation and apoptosis in the collected cancer tissues post **M1-20** treatment (Fig. 4P). Furthermore, we noticed that the protein levels of cancer stem cell markers ALDH1 and CD44 were also decreased in the **M1-20**-treated cancer tissues (Supplemental Fig. 6H), implicating that the population of cancer stem cells in the grafted cancers were altered by the **M1-20** treatment.

### M1-20 inhibited FOXM1-related transcriptional activities

**Fig. 5:**
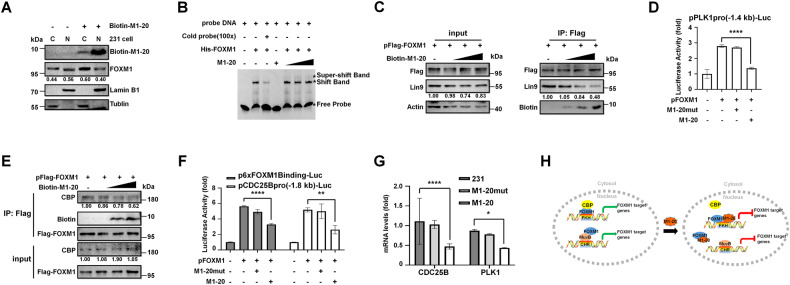
M1-20 inhibited FOXM1-related transcriptional activities. **A** MDA-MB-231 cells were seeded in 6 cm plates for 12 h and then treated with **M1-20** (10 µM) containing 10% biotin-labeled **M1-20**. After treatment for 24 h, the cells were harvested for the separation of the cytoplasmic and nuclear proteins. The levels of **M1-20** in the cytoplasm (C) or the nucleus (N) were analyzed by Western blotting. α-Tubulin or Lamin B1 was used as a cytoplasmic or nuclear marker, respectively. **B** The EMSA experiment was performed by recombinant His-FOXM1 proteins (2 µM) and the FAM-labeled DNA probe (50 nM). **M1-20** was added to the reactions with increasing concentrations (5 µM and 10 µM). The Cold probe (100×, 5 mM) was unlabeled with FAM to show the specificity of FOXM1/DNA complex formation. The reactions were performed in 4% native polyacrylamide gel electrophoresis in 0.5×TBE buffer and imaged with Kodak 4000 MM Imaging System (EX: 465 nm, EM: 535 nm for FAM). **C** Hela cells were transfected with pFlag-FOXM1 (6 µg) for 48 h. Cell lysates were harvested and respectively incubated with Flag magnetic beads, added to different dosages of biotin-labeled **M1-20** (0 μg, 2 μg, 4 μg, 8 μg). Flag-FOXM1/protein complexes were analyzed by Western Blotting with certain antibodies. 10% of cell lysates (50 μg) were used as input controls. The levels of protein were quantified by Image J software. **D** The −1.4 kb PLK1 promoter-luciferase reporter plasmid (1 µg) and pRL-CMV plasmid (20 ng/well) were co-transfected with pFOXM1 (0.3 µg) into Hela cells. 12 h later **M1-20** (20 µM), and **M1-20mut** (20 µM) were respectively added to the selected transfections. Then cell lysates were prepared 24 h later and used for the measurement of dual Luciferase activities. *n* = 3 for each group, ****P < 0.0001, two-tailed unpaired Student’s t-test. **E** HEK293T cells were transfected with pFlag-FOXM1 (6 µg) and 48 h later the cells were collected. The cell lysates (500 μg) were incubated with Flag magnetic beads and added with different quality of **M1-20** (0 μg, 2 μg, 4 μg, 8 μg) to immunoprecipitation Flag-FOXM1/protein complexes. CBP, Biotin, and Flag proteins in samples were detected by Western Blotting with certain antibodies. 10% of cell lysates (50 μg) were used as input controls. **F** Reporter plasmids containing the 6×FOXM1 binding sequence (1 µg) or the −1.8 kb CDC25B promoter-luciferase reporter (1 µg) were co-transfected with pFOXM1 (0.3 µg) into HEK293T cells, plus pRL-CMV plasmid (20 ng/well) as a loading control. 12 h later **M1-20** (20 µM) or **M1-20mut** (20 µM) was added to the selected transfections. Another incubation for 24 h later cell lysates were prepared for the measurement of dual Luciferase activities. *n* = 3 for each group, **P < 0.01, two-tailed unpaired Student’s t-test. **G** MDA-MB-231 cells were treated with **M1-20** (10 µM) or **M1-20mut** (10 µM) and 24 h later the cells were collected for the preparation of total RNA. The mRNA levels of CDC25B and PLK1 were examined by RT-RCR. *n* = 3 for each group, ****P ≤ 0.0001, two-tailed unpaired Student’s t-test. **H** The diagram depicting the molecular mechanisms of **M1-20** inhibiting the FOXM1-related transcriptional activities.

Although it was detected in both cytoplasm and nucleus, **M1-20** majorly localized in the nucleus of cells (Fig. 5A). Interestingly, **M1-20** also affected the distribution of FOXM1 in cells, resulting in the elevated levels of FOXM1 in cytoplasm and the declined levels of FOXM1 in nucleus (Fig. 5A). Electrophoretic Mobility Shift Assays (EMSAs) showed that **M1-20** did bind to FOXM1 but not disrupt its DNA binding ability (Fig. 5B). Because **M1-20** bound to FOXM1 N-terminus that interacted with the MuvB complex [27], we asked whether **M1-20** affected the interaction between FOXM1 and the key component of the MuvB complex, LIN9, which recruited FOXM1 and bound to CHR-containing promoters, such as the promoter of PLK1 [28]. We performed Co-IP experiments to verify that the interaction of FOXM1:LIN9 was disrupted in cells by **M1-20** in a dose-dependent manner (Fig. 5C). This was further supported by co-transfection experiments, in which **M1-20** abolished the FOXM1-MuvB-mediated stimulation on the −1.4 kb PLK1 promoter (Fig. 5D), similar to the effects of the LIN9 siRNA treatment (Supplemental Fig. 7). In addition, because the transcriptional activation of FOXM1 on FKH-containing promoters relied on recruiting transcriptional co-activator CBP at FOXM1 C-terminus [40], we verified that **M1-20** disrupted the interaction between FOXM1 and CBP by Co-IP experiments (Fig. 5E). **M1-20** abolished the FOXM1-mediated stimulation on the FKH-containing promoters in co-transfection experiments (Fig. 5F). Finally, we measured the mRNA levels of FOXM1 typical target genes, CDC25B and PLK1, and observed the downregulated levels of both genes in MDA-MB-231 cells post **M1-20** treatment (10 μM for 24 h) (Fig. 5G), further supporting the molecular mechanisms summarized in a diagram (Fig. 5H) for **M1-20** inhibiting FOXM1-related transcriptional activities.

### M1-20 inhibited the progression of cancers in wild-type mice

**Fig. 6:**
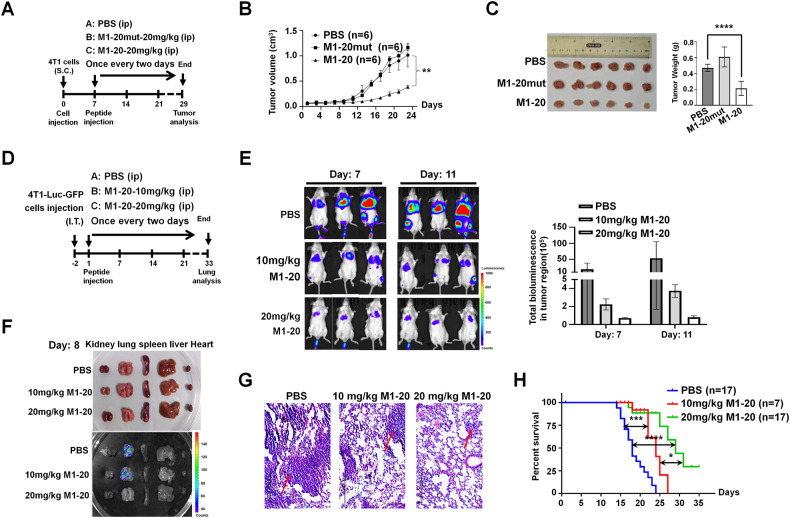
M1-20 inhibited the progression of cancers in wild-type mice. **A** BALB/c mice (female, 4–6 weeks old) were subcutaneously (S.C.) injected with 4T1 cells (1 × 10^6^ cells/injection) into the left and right flank. One week later, the mice were randomly divided into three groups (three mice/group) and intraperitoneally (I.P.) injected with PBS, **M1-20mut** (20 mg/kg) or **M1-20** (20 mg/kg) for three weeks. **B** Tumor volume was measured every two days and growth curves were obtained at the end of the experiment. Tumor volume (*V*) was calculated as: *V* = length × diameter^2^ × 1/2. Significant differences were shown, 2-way ANOVA (Sidak’s multiple-comparisons test): **P ≤ 0.01. **C** The images and weight of engrafted tumors at the end of the experiment (****P ≤ 0.0001). **D** BALB/c mice (female, 4–6 weeks old) were injected intravenously with 4T1-Luc-GFP cells (5 × 10^4^ cells/mouse). After three days, the mice were randomized into three groups and injected (I.P.) with PBS, **M1-20** (10 mg/kg, 20 mg/kg) every two days. **E** The growth of metastatic 4T1-Luc-GFP cancer cells was observed on Day 7 and Day 11 with the intraperitoneal injection of D-Luciferin potassium salt (3 mg/200 μL/mouse) through whole-body bioluminescence imaging by IVIS Lumina XR. Fluorescence intensities in three mice selected randomly were measured by the Living Image^®^ software. **F** Ex vivo fluorescence images of different organs on Day 8 after **M1-20** treatment were taken using IVIS Lumina XR. **G** The status of metastatic lung cancer tissue on Day 8 was performed by H&E staining. Representative images were shown. Scale bar: 20 μm. **H** Survival statistics for the mice were graphed using GraphPad Prism 9 software with Mantel-Cox estimator and log-rank test (*P ≤ 0.05, **P ≤ 0.01, ***P ≤ 0.001, ****P ≤ 0.0001).

Next, mouse cancer models with wild-type backgrounds and intact immune systems were used to analyze **M1-20** anti-cancer effects in vivo. Both domains of human FOXM1 (1-138aa and 689-748aa) were highly conserved with those of mouse Foxm1 (Supplemental Fig. 8A). Biotin-labeled **M1-20** could bind to endogenous Foxm1 from mouse breast cancer 4T1 cell lysates (Supplemental Fig. 8B) and as expected, **M1-20** inhibited 4T1 cells at a dose-dependent manner (Supplemental Fig. 8C). 4T1 cells (1 × 10^6^ cells/injection) were subcutaneously implanted in the left and right flanks of wild-type BALB/c mice (Fig. 6A). Compared with the PBS and **M1-20mut** groups, the intraperitoneal injection of **M1-20** resulted in the inhibition of cancer growth (Fig. 6B), and significantly reduced the size and weight of engrafted cancers (Fig. 6C), corresponding to the decreased levels of proliferation-related CDC25B, PLK1, PCNA, and increased levels of apoptosis-related Bax and Caspase 3 in **M1-20**-treated cancer samples (Supplemental Fig. 9A, B). Immunostaining showed that the levels of PCNA were downregulated and the levels of Caspase 3 were upregulated post the **M1-20** treatment (Supplemental Fig. 9C). Our results demonstrated that systemic administration of **M1-20** exhibited a strong anti-cancer effect in wild-type animals.

To investigate the impact of **M1-20** on the metastasis of cancer cells in wild-type mice, we constructed a stable luciferase-GFP-expressed cell line with 4T1 cells (4T1-Luc-GFP) (Supplemental Fig. 10). 4T1-Luc-GFP cells (5 × 10^5^ cells/mouse) were injected into BALB/c mice (females, 4–6 weeks of age) via tail-vein (Fig. 6D). Therapeutic efficacy was monitored by bioluminescence imaging, which was performed by randomly selecting three mice with the intraperitoneal injection of D-Luciferin potassium salt (3 mg/200 μL/mouse) at different time points after **M1-20** systemic administration (Day 7 and Day 11). Bioluminescent signals showed that **M1-20** prevented the metastasis of cancer cells in a dose-dependent manner (Fig. 6E). One mouse was randomly taken from each group on Day 8 to analyze metastatic cancer cells in different organs. Lumina imaging and H&E staining revealed that **M1-20** inhibited the metastasis of cancer cells mainly to the lung (Fig. 6F, G). The expression levels of migration-related genes in metastatic cancer cells of lung tissue were also altered by **M1-20** treatment (Supplemental Fig. 11). The survival of animals was monitored throughout the experiments, with the survival curve illustrating that **M1-20** significantly prolonged survival time in a dose-dependent manner (Fig. 6H). These results demonstrated that systemic administration of **M1-20** showed a potent anti-metastasis effect on cancer cells and improved the survival of animals.

To evaluate the safety of **M1-20**, H&E staining on tissue sections from BALB/c mice treated with **M1-20** showed no obvious morphological lesions in multiple organs (Supplemental Fig. 12A). Hemolysis analysis demonstrated that **M1-20** with a concentration gradient (25, 50, 100, 200, 400, 800 µg/ml) incubated with blood cells did not induce obvious hemolysis (Supplemental Fig. 12B). Acute toxicity test results showed that wild type ICR/JCL mice [41, 42] could tolerate doses of **M1-20** up to 200 mg/kg body weight by intraperitoneal injection with no observed toxicity (Supplemental Fig. 12C). ELISA assays showed no significant increase in the generation of **M1-20**-specific antibodies at the time points tested compared with pre-**M1-20** injection (Supplemental Fig. 12D). Together, these results showed that **M1-20** was well-tolerated and safe at its dose of anti-cancer therapy in vivo.

## Discussion

Peptides have many favorable characteristics, such as moderate molecular weight, high binding affinities, significant target specificities, and relatively safe and well-tolerated, which have garnered much attention for the development of anti-cancer drugs [3]. In particular, interfering peptides possess advantages for disrupting transcription factor-involved PPIs due to the large and flat contact surfaces. In this study, we have developed a novel FOXM1-interfering peptide **M1-20** that potently represses FOXM1 functions by disrupting PPIs between FOXM1 and its multiple partner proteins.

**M1-20** binds to the N-terminal domain of FOXM1 with relatively high affinity (K_D_ = 5.658 μM) to disrupt the interaction between FOXM1 and the MuvB complex, thereby inhibiting FOXM1 transcriptional activities on certain cell cycle genes such as PLK1. Furthermore, we have noted that **M1-20** can abolish the interaction between FOXM1 and CBP without affecting FOXM1’s DNA-binding ability, explaining the inhibition of FOXM1 direct downstream genes such as CDC25B. In addition, FOXM1 activity is induced by the phosphorylation of multiple sites in its C-terminal domain by cell cycle-related protein kinases during mitosis, such as CDK1 [30] and PLK1 [40], which are essential protein kinases in G2/M phase to stimulate cell cycle progression and are considered as therapeutic targets in various cancers [43]. Interestingly, both kinases appear on the list of **M1-20**-interacting candidates from our ongoing Mass Spectrometry Analysis with high confidence (data not shown). Therefore, it is worth testing whether **M1-20** acts as an inhibitor for both kinases in future studies.

Generally, natural peptides (composed of natural L-amino acids) are highly susceptible to protease degradation, resulting in poor stability and limited therapeutic potential [39]. The development of DRI peptides, which incorporate D-amino acids as stable surrogates of L-amino acids but are assembled in a reverse (retro) order compared with the parent peptide [44], provides an efficient approach to overcome these limitations. Since their side chains adopt a topology similar to that of the parent peptide [45], DRI peptides have the potential to achieve the same functions as their parent L-peptides with superior stability towards proteolytic degradation [44]. Several DRI peptides have been evaluated as anti-tumor drug candidates in preclinical studies, e.g., DRI peptide RE-A7R targeting VEGFR2 [46], FOXO4-DRI peptide targeting p53:FOXO4 interaction [47], or in clinical trials, e.g., AM111 targeting JNK:c-Jun interaction [48]. In this study, **M1-20**, the DRI form of the **P5** peptide, possesses a prolonged half-life in cell lysates and exhibits a conformation similar to **P5**. The binding affinity and inhibitory potency of **M1-20** to cancer cells are dramatically improved, indicating the successful optimization of the peptide using the DRI strategy. In addition, many other approaches have been proposed to optimize peptides, including amino acid substitution, cyclization, side chain stapling, PEGylation, or lipidation [49], which can be explored for the selected **P5** peptide in the future.

Although **M1-20** has been primarily studied for its anti-tumor effects in breast cancer models, it has the potential to inhibit various types of cancer by targeting the FOXM1 protein. The levels of FOXM1 are elevated in almost all clinical cancer types from the TCGA database [50]. Conditional knockout of FOXM1 inhibits cancer development in multiple mouse organs, such as the liver [51], lung [52], and rectum [53]. Our studies have also demonstrated that adenovirus-mediated interference with FOXM1 expression impedes the progression of liver cancer [54], breast cancer [13], and nasopharyngeal carcinoma [55]. While **M1-20** has been shown to inhibit various cancer cell lines in cell culture, its effects on different solid tumors in vivo require further investigation. Furthermore, **M1-20** may have potential benefits in cancer treatment when combined with standard chemotherapy or radiotherapy. Recently, studies have demonstrated the involvement of FOXM1 in chemoresistance [12], and several FOXM1-targeting small molecule compounds, such as Siomycin A plus 5-FU [22], Thiostrepton plus Selumetinib [56], and FDI-6 plus PARP inhibitor Olaparib [57], have been tested in combination therapy to overcome drug resistance in cancer treatment. These combinations have been explored as a solution to the narrow clinical indications and adaptive resistance of FOXM1 inhibitors. As peptides display minimal susceptibility to drug resistance [58], it is possible that combining **M1-20** with standard chemoradiotherapy could improve the efficacy of cancer treatment.

This study focuses on the therapeutic targeting of FOXM1 with **M1-20**, with the aim of altering cancer cell phenotypes such as proliferation, migration, and apoptosis. Notably, treatment with **M1-20** leads to down-regulation of cancer stem cell markers including ALDH1 and CD44 (Supplemental Fig. 6H), suggesting its potential in modulating cancer stem cell populations. Given that FOXM1 plays a crucial role in maintaining cancer stem cell properties [15], further studies should explore the effects of **M1-20** on cancer stem cell populations during initial and recurring stages of cancer. Such evidence could inform optimal therapeutic timing of **M1-20** in clinical settings. Overall, our study provides valuable insights into the therapeutic effects of **M1-20** against cancers and suggests that it may be a promising candidate for further clinical investigation.

## Supplementary information

Supplemental Material and Figures

Original WB data

Reproducibility checklist

## Data Availability

All data generated or analyzed during this study are included in this manuscript and its Supplementary Information files. Additional data are available from the corresponding author upon reasonable request.
