# Supplementary material for: Development of an interfering peptide M1-20 with potent anti-cancer effects by targeting FOXM1
Source: Cell Death Dis. 2023 Aug 19;14(8):533. doi: 10.1038/s41419-023-06056-9 (PMC10439915; doi:10.1038/s41419-023-06056-9)
Supplement: Supplementary file 1 — Supplemental Material and Figures [file 41419_2023_6056_MOESM1_ESM.docx]

**Supplementary data to Bu et al. “Development of an interfering peptide M1-20 with potent anti-cancer effects by targeting FOXM1”**

Table of Contents

**Supplementary figures**4

Figure S1. FOXM1_689-748_ was required for FOXM1 transcriptional activity and interacted with FOXM14

Figure S2. The analysis of peptides **P5** and **P5mut** by MALDI TOF MS and HPLC5

Figure S3. The analysis of peptides **M1-20 and M1-20mut** by MALDI TOF MS and HPLC6

Figure S4. Sequence and secondary structure comparison of **P5** and **M1-20**7

Figure S5. The dependency of **M1-20** anti-cancer efficiency on the levels of FOXM1 in cancer cells.8

Figure S6. **M1-20** inhibited the proliferation and migration of cancer cells, and induced apoptosis of cancer cells9

Figure S7. The inhibition of **M1-20** on FOXM1 transcriptional activity was similar to LIN9 siRNA treatment11

Figure S8. **M1-20** bound to mouse Foxm1 and inhibited mouse breast cancer 4T1 cells12

Figure S9. **M1-20** inhibited proliferation and induced apoptosis of 4T1 cell-engrafted cancer tissue13

Figure S10. The construction of 4T1-Luc-GFP cells14

Figure S11. **M1-20** treatment altered migration-related gene expression levels of metastatic cancers in lung15

Figure S12. **M1-20** was well tolerated in *vivo*16

**Supplementary methods**18

Construction of plasmids18

Expression and purification of recombinant proteins20

Cell culture20

Luciferase reporter assay20

Solid-phase synthesis of peptides21

Structural modeling with Rosetta FlexPepDock21

Microscale thermophoresis assay22

Pull-down and Co-immunoprecipitation (Co-IP) Assays23

RNA Sequencing23

Protein extraction and Western blotting23

RNA isolation and real-time quantitative PCR (RT-PCR)24

Confocal imaging25

Lentivirus construction and infection25

Cell proliferation assay25

Cell cycle analysis26

Wound-healing assay26

Apoptosis assay27

Electrophoretic mobility shift assays (EMSAs)27

The anti-cancer effects of **M1-20** *in vivo*28

Immunohistochemistry29

Histological and morphological analyses30

Hemolysis assays30

Toxicity test and immunogenicity assays of **M1-20**31

Statistical analysis31

**References**32

**Supplementary tables**35

Supplementary table S133

Supplementary table S235

**

**

**Supplementary Figure 1. FOXM1_689-748_ was required for FOXM1 transcriptional activity and interacted with FOXM1.**

(**A**) The reporter plasmid, containing 6×FOXM1 binding sequences in its promoter (p6×FOXM1Binding-Luc, 1 µg) was transfected with pFOXM1 (0.3 µg) or pFOXM1_1-688_ (0.3 µg) into HEK293T cells, plus pRL-CMV plasmid (20 ng/well) as a loading control. After 48 h, cell lysates were collected and prepared for the measurement of dual Luciferase activity. n = 3 for each group, ***P* < 0.01, two-tailed unpaired Student’s t-test. (**B**) MDA-MB-231 cell lysates (500 μg) were prepared, and respectively incubated with GST protein (50 μg) or GST-FOXM1_689-748_ protein (50 μg) at 4℃, added with GST-tag resin. FOXM1 and GST-tag antibodies were used for Western Blotting. 10% of cell lysates (50 μg) were used as input controls.

**
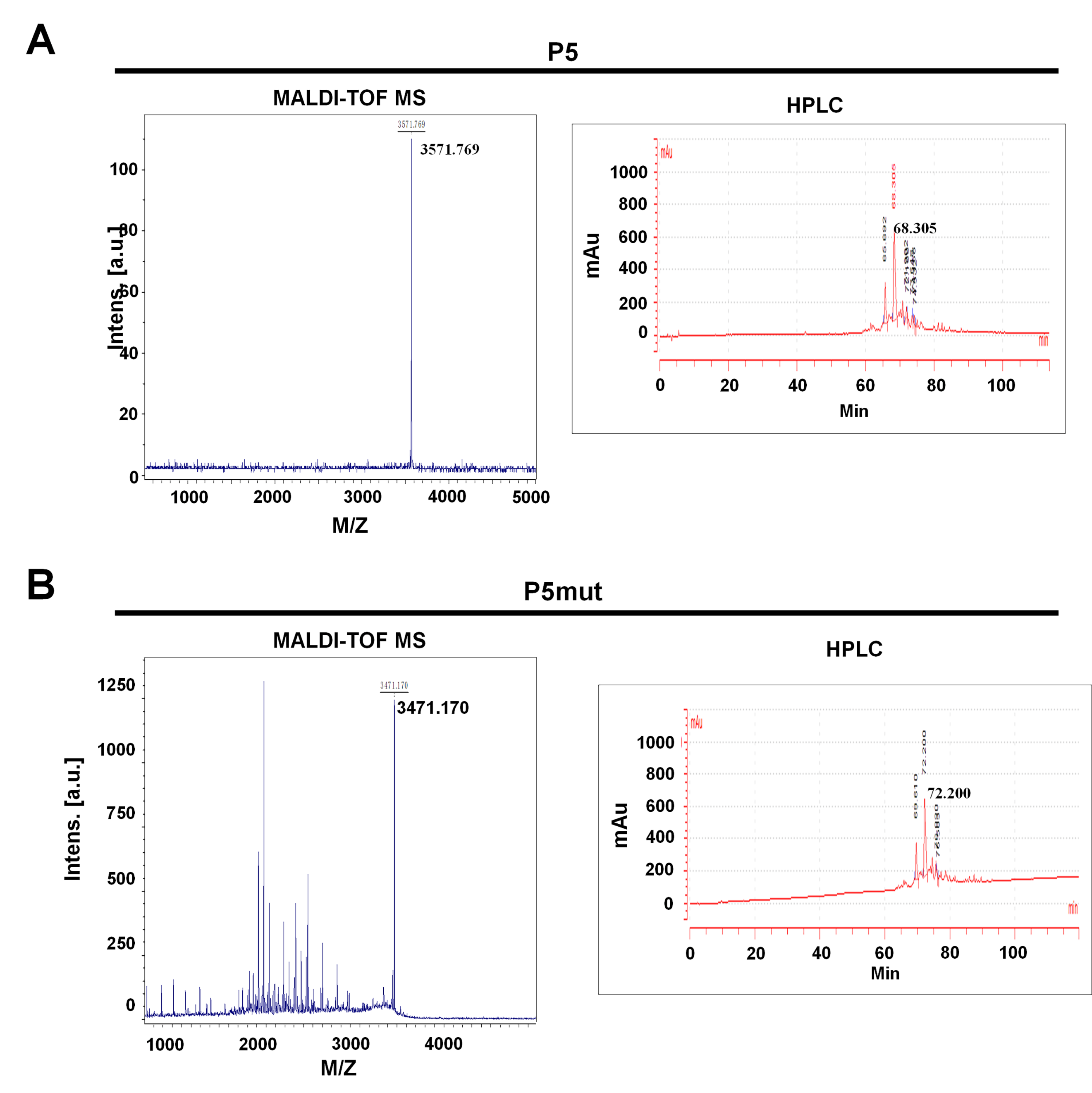
**

**Supplementary Figure 2. The analysis of peptides P5 and P5mut by MALDI TOF MS and HPLC.**

(**A**-**B**) The molecular weight of the peptide was measured using Matrix-Assisted Laser Desorption/Ionization Time of Flight Mass Spectrometry (UltrafleXtreme, BRUKER, Germany). The purity of the peptide was determined by Reversed Phase High-Performance Liquid Chromatography (LC-2010&LC1010, RAINBOW, China) with acetonitrile and water (0.05%TFA) as mobile phase and the C18 column (kromasil 100-5 C18 250*4.6*5, Sweden). At the elution time (90 min), the elution gradient (0.5% to 70% acetonitrile) was set for the separation of the peptide.

**
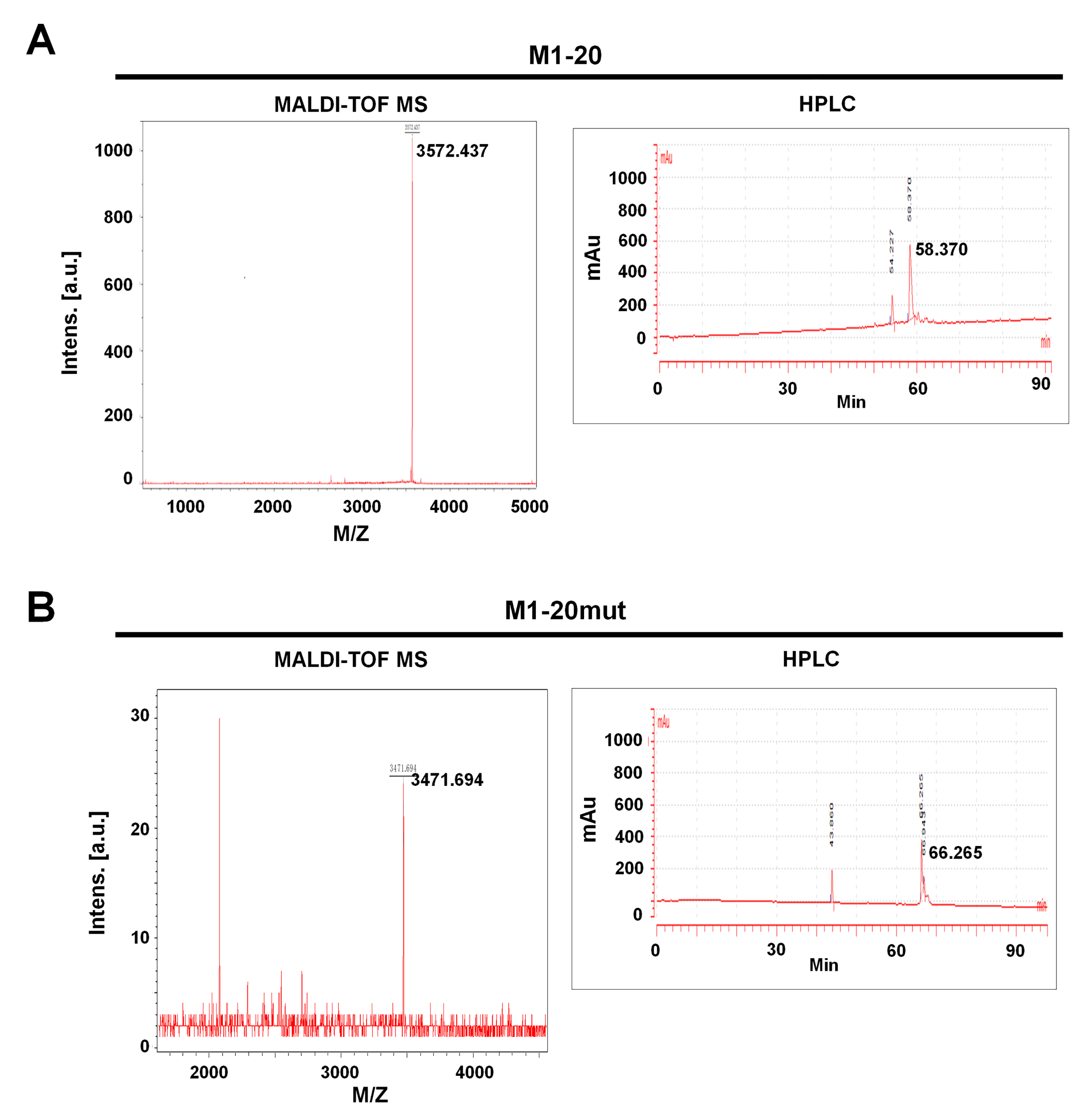
**

**Supplementary Figure 3. The analysis of peptides M1-20 and M1-20mut by MALDI TOF MS and HPLC.**

The methods were the same as in Supplementary Figure 2.

**
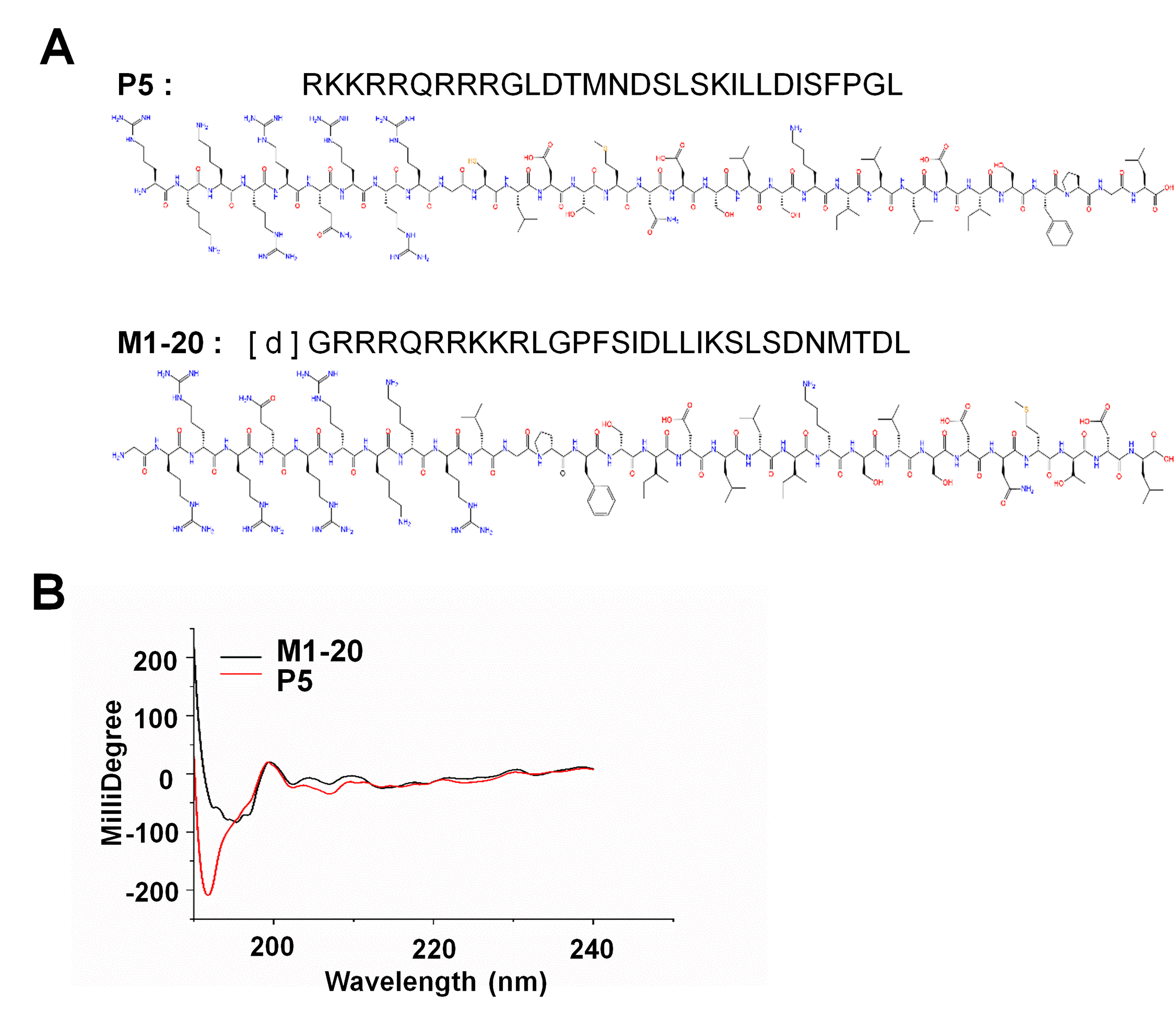
Supplementary Figure 4. Sequence and secondary structure comparison of P5 and M1-20.** (**A**) Comparison of sequences and branched-chain structures between **P5** and **M1-20.** (**B**) Peptides (5 μm) were dissolved in phosphate-buffered saline (PBS) containing 30% TFE (2, 2, 2-Trifluoroethanol) and analyzed using a Bio-Logic MOS-500 CD spectrometer with a 1-mm path-length quartz cuvette. The CD spectra were scanned in the range of 190 to 240 nm with PBS alone getting blank and analyzed using Origin 2023.

**

**

**Supplementary Figure 5. The dependency of M1-20 anti-cancer efficiency on the levels of FOXM1 in cancer cells.** (**A**) FOXM1 expression in MBA-MD-231 (231), U2OS, ZR-75-30, MCF7, A549, Hela, MCF10A, and HUVEC cells was detected by Western blotting. Correlation analysis of the cytotoxic effect of **M1-20** and the protein levels of FOXM1. Person’s correlation was used for analyzing the data (**P* < 0.05). (**B-C**) The protein levels of FOXM1 in MCF-7 (**B**), Hela (**C**), and FOXM1-overexpressing cell lines (MCF7-OE, Hela-OE) were measured by Western blotting. Cells were seeded in 96-well plates for 12 h and treated with a defined concentration gradient of **M1-20** or **M1-20mut** (0, 10, 20, 40, 60, 80 µM). 36 h later, CCK-8 solution (10%) was added to each well and incubated for another 2 h. The absorbance at 450 nm was measured, and each well's relative cell viability was calculated (n=3).


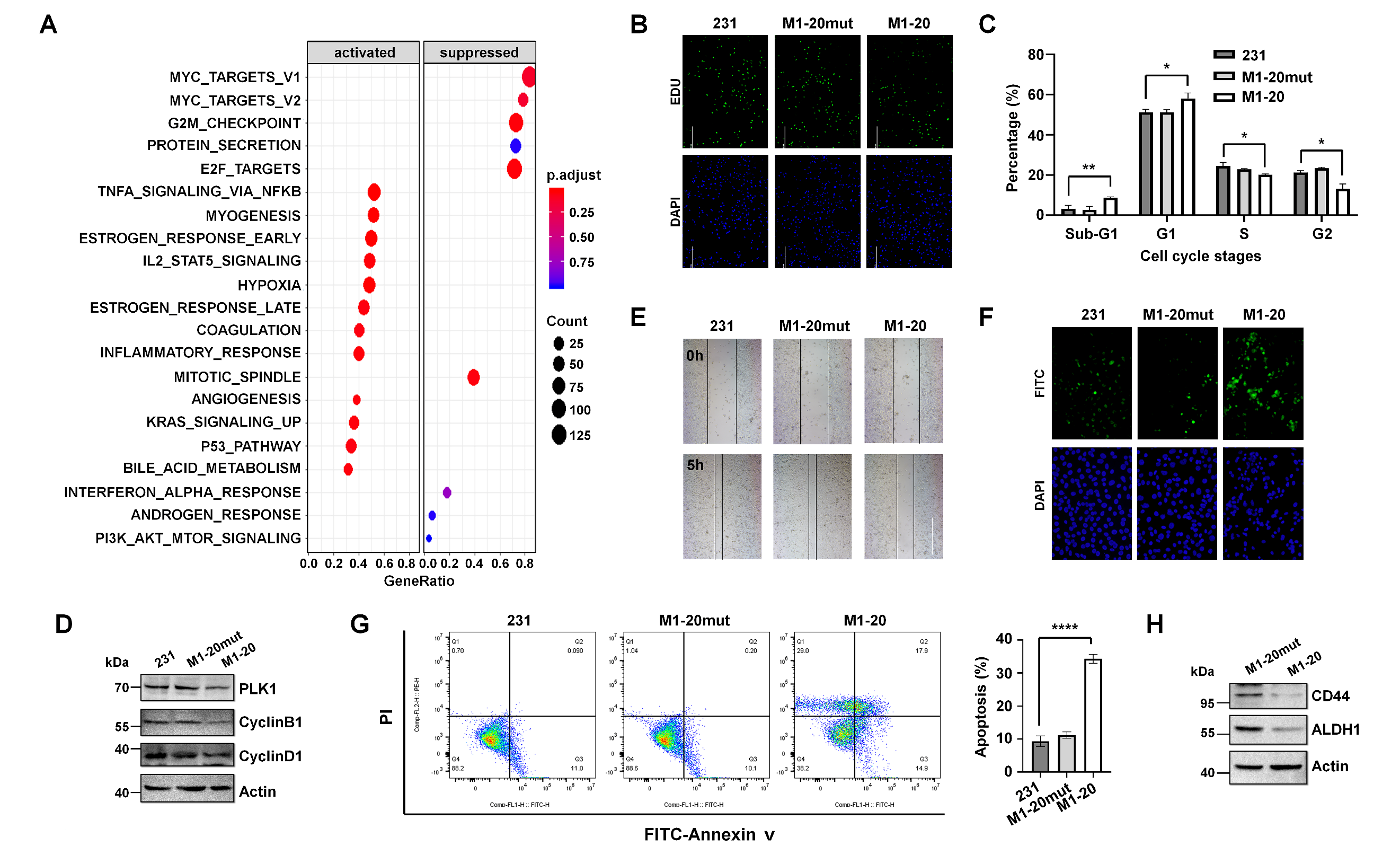


**Supplementary Figure 6.** **M1-20 inhibited the proliferation and migration of cancer cells, and induced apoptosis of cancer cells.** (**A**) MDA-MB-231 cells were treated with **M1-20** (10 µM) or **M1-20mut** (10 µM) and 24 h later the cells were extracted the total RNA for RNA sequencing. Hallmark signaling pathways within the Molecular Signatures Database (MSigDB, https://www.gsea-msigdb.org/gsea/msigdb/) were significantly enriched using the differentially expressed genes (DEGs) based on the gene set enrichment analysis (GSEA). (**B**) MDA-MB-231 cells (2 × 10^4^ cells/well) were seeded in 24-well plates and respectively treated with **M1-20** (10 µm), **M1-20mut** (10 µm), or non-treated for 24 h. Cells were incubated with EdU for another 2 h. After fixing and permeating, cells were treated with 100µl Click Reaction Mixture for 30 min in the dark at room temperature. The nucleus was stained with Hoechst 33342 for 20 min and photographed with a fluorescence microscope (40×, Nikon TE2000). Scale bar: 100 μm. (**C**) MDA-MB-231 cells were treated with **M1-20** (10 µM) and 24 h later the cells were harvested for PI staining. Cell cycle of MDA-MB-231 cells was analyzed by Flowcytometry. n=3, **P* < 0.05, ***P* < 0.01. (**D**) MDA-MB-231 cells were treated as Figure 4C. The protein levels of PLK1, Cyclin B1 and Cyclin D1 were measured by Western Blotting. (**E**) MDA-MB-231 were seeded in a 12-well plate at 2.8 × 10^5^ cells per well. A 200 μl pipette tip was used to scratch a line when cells achieved a 100% confluent monolayer. Cells were photographed at 0 h and treated with **M1-20** (10 µM) or **M1-20mut** (10 µM) for a moment. Migration was monitored at different times after wound formation. Scale bar: 1 000 μm. (**F**) MDA-MB-231 cells were seeded in a 12-well plate and respectively treated with **M1-20** (10 µm), **M1-20mut** (10 µm), or non-treated for 24 h. Then, cells were fixed with 4% paraformaldehyde for 30 min and incubated with 50µl TUNEL detection mixture for 1h at room temperature without light. The location of the nucleus was stained by DAPI. (**G**) MDA-MB-231 cells were treated with **M1-20 (**10 µM**)** and 24 h later were harvested for cell apoptosis analysis by Flow cytometry. (**H**) The protein levels of CD44 and ALDH1 in collected tumor tissues were measured by Western Blotting.

**
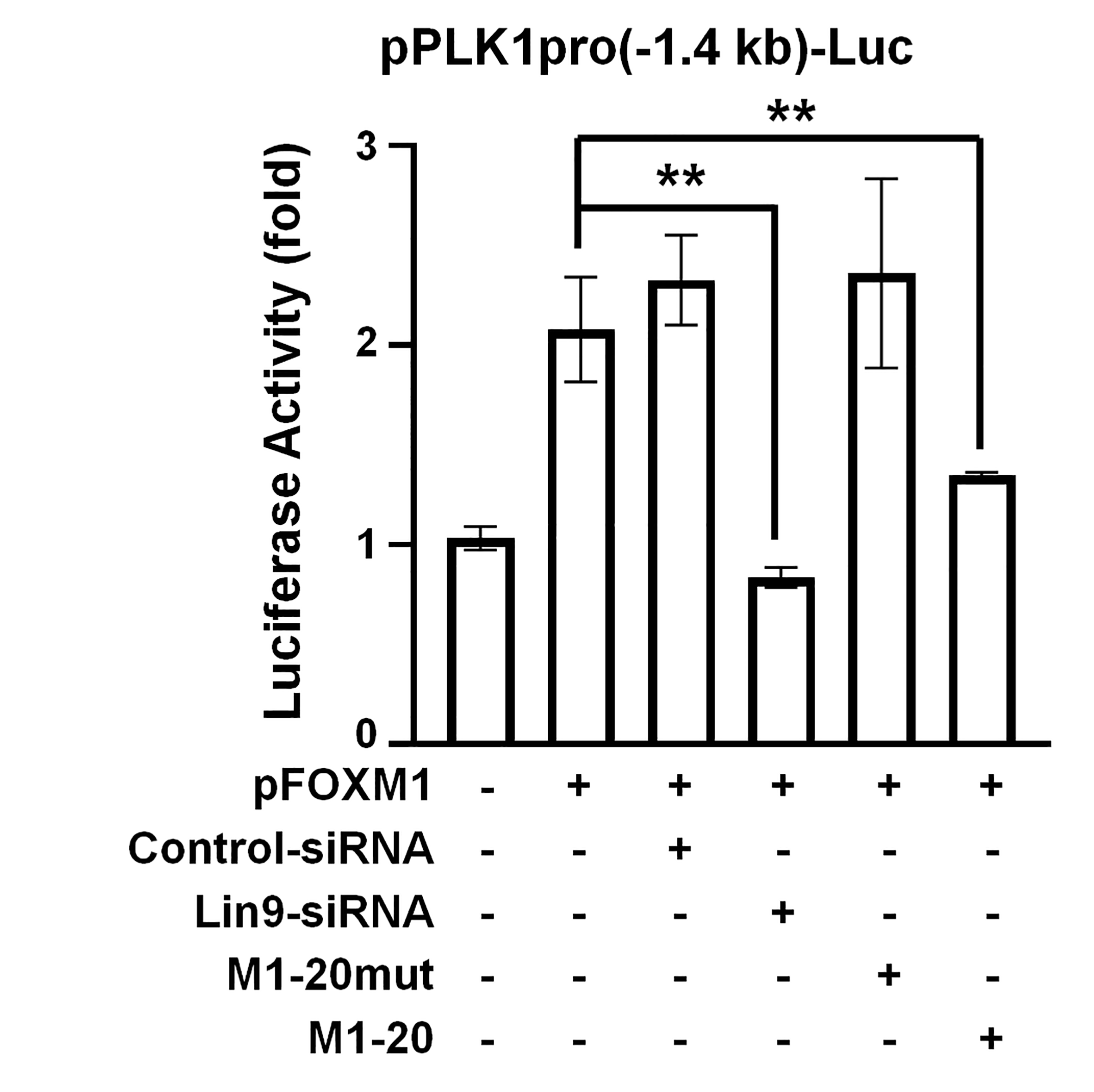
**

**Supplementary Figure 7.** **The inhibition of M1-20 on FOXM1 transcriptional activity was similar to LIN9 siRNA treatment.**

The FOXM1 expression vector (pFOXM1, 0.3 μg) was transfected with the -1.4 kb PLK1 promoter-luciferase reporter plasmid (1 μg) and pRL-CMV plasmid (20 ng) into Hela cells, and Contrlo-siRNA, LIN9-siRNA was respectively transfected to the corresponding groups. 12 h later, **M1-20** (10 µM) and **M1-20mut** (10 µM) were added to the selected groups. After another 24 h, protein lysates were prepared and used for the measurement of dual Luciferase activity. n = 3 for each group, ***P* < 0.01, two-tailed unpaired Student’s t-test.

**
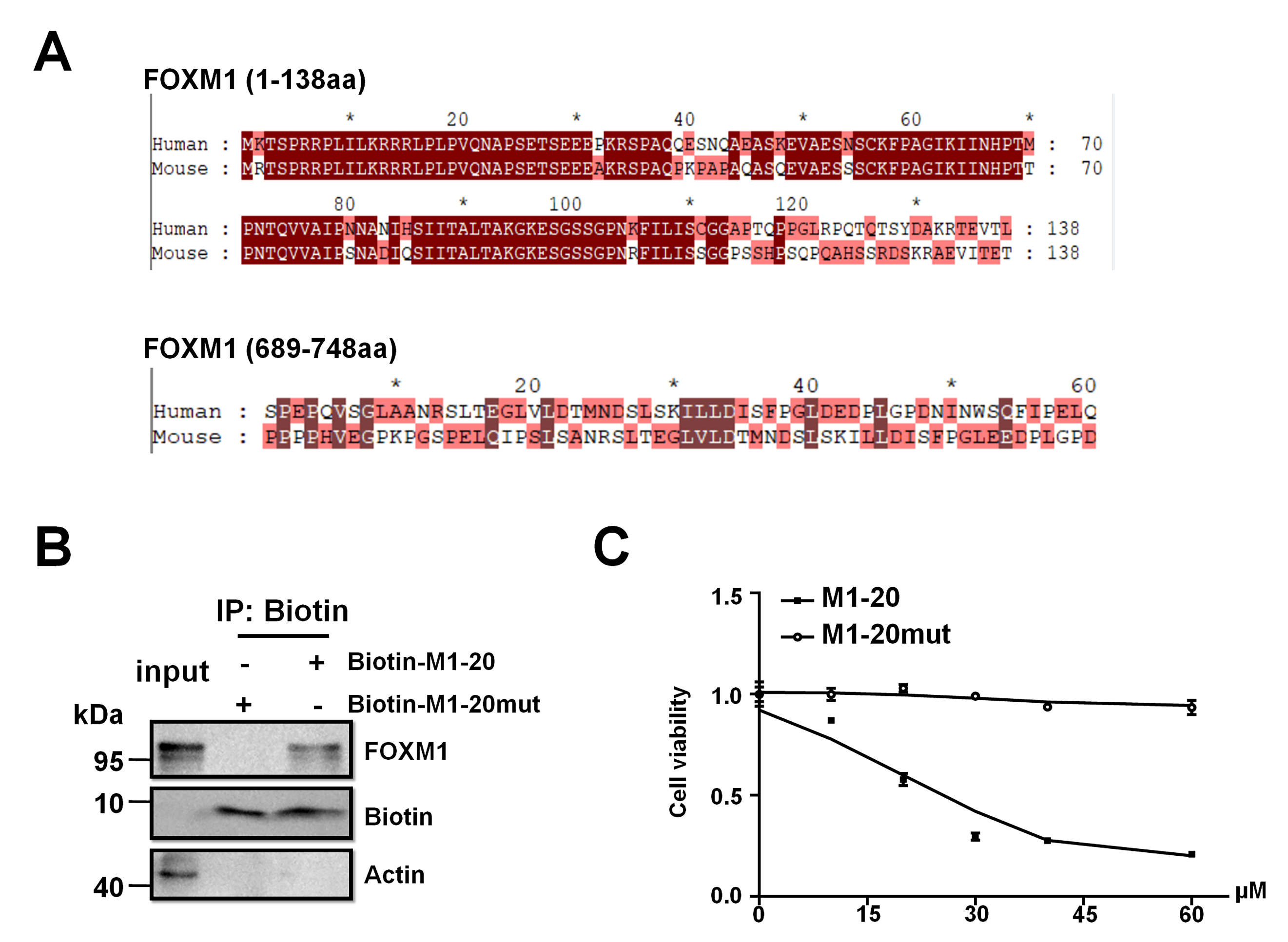
**

**Supplementary Figure 8.** **M1-20 bound to mouse Foxm1 and inhibited mouse breast cancer 4T1 cells.** (**A**) Sequence alignment between different fragments (1-138aa, 689-748aa) of FOXM1 from human and mouse. (**B**) Biotin-labeled **M1-20** (**Biotin-M1-20**) or **M1-20mut** (**Biotin-M1-20mut**) was incubated with 4T1 cell lysates (500 µg), added to Streptavidin Magnetic Beads. Biotin-peptide/protein complexes were detected by Western blotting. 10% of cell lysates (25 µg) were used as input controls. (**C**) 4T1 cells (4×10^3^ cells/well) were seeded in 96-well plates for 12 h and treated with a defined concentration gradient of **M1-20** or **M1-20mut** (0, 10, 20, 30, 40, 60 µM). 36 h later, CCK-8 solution (10%) was added to each well and incubated for another 2 h. The absorbance at 450 nm was measured, and each well's relative cell viability was calculated (n=3).

**
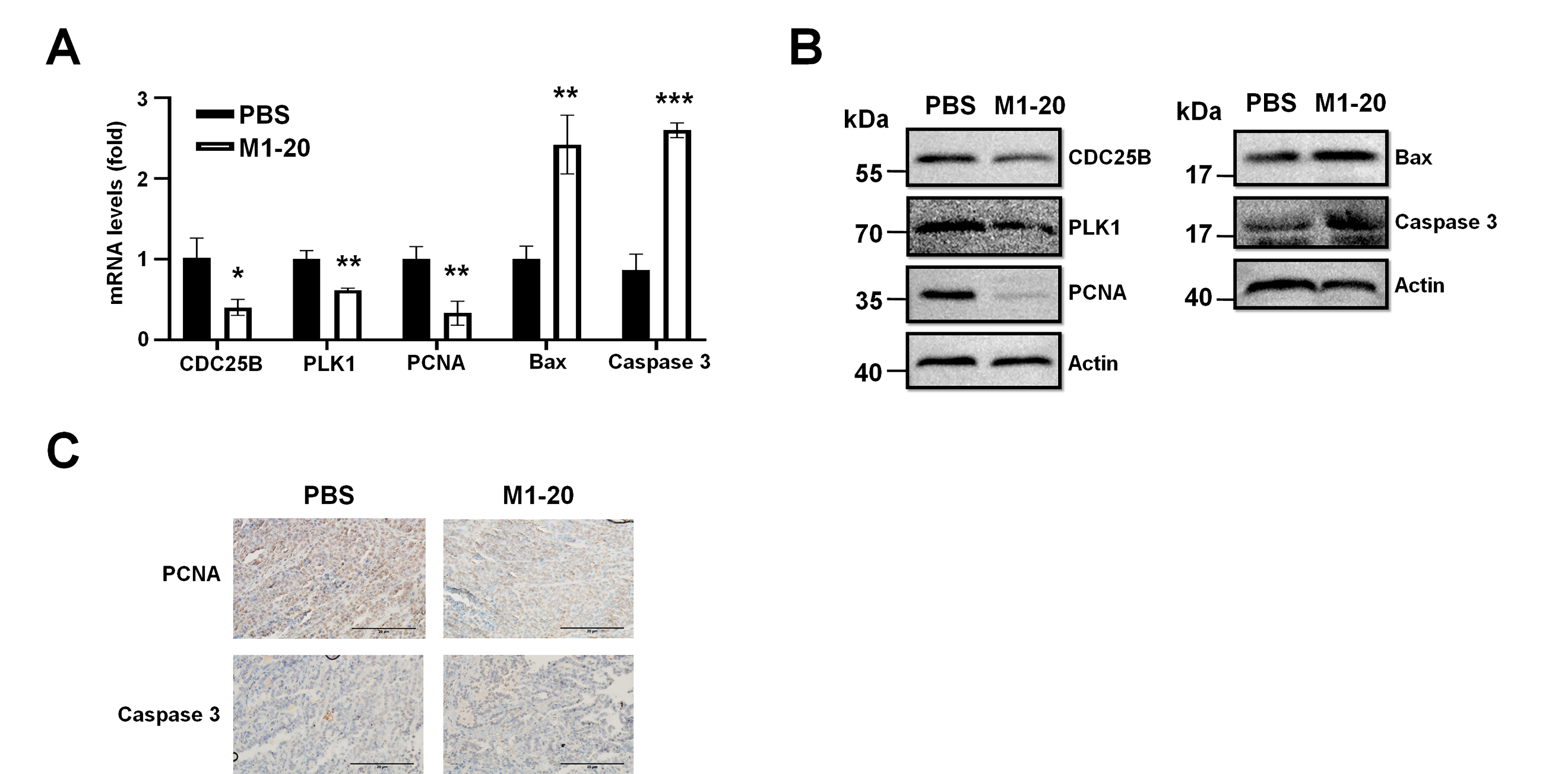
**

**Supplementary Figure 9.** **M1-20 inhibited proliferation and induced apoptosis of 4T1 cell-engrafted cancer tissue.** (**A**-**B**) Total RNA (**A**) and total proteins (**B**) after **M1-20** treatment were prepared with harvested cancers. Total RNA samples from both groups were extracted with Trizol reagent. The mRNA levels of CDC25B, PLK1, PCNA, Bax, and Caspase 3 were examined by RT-PCR. Relative mRNA levels were normalized to GAPDH. n = 3 for each group, **P* ≤ 0.05, ***P* ≤ 0.01, ****P* ≤ 0.001, two-tailed unpaired Student’s t-test. After homogenization and grinding of tumor tissue, tumor cells were lysed with RIPA lysis and extraction buffer to obtain whole cell extracts. Protein levels were measured by Western blotting with certain antibodies. (**C**) Tumor tissue slices from the groups were immunostained with PCNA or Caspase 3 antibody (antibody dilution ratio was 1:100). Photographs were taken with Olympus IX-73 inverted microscope. Scale bar: 20 μm.

**
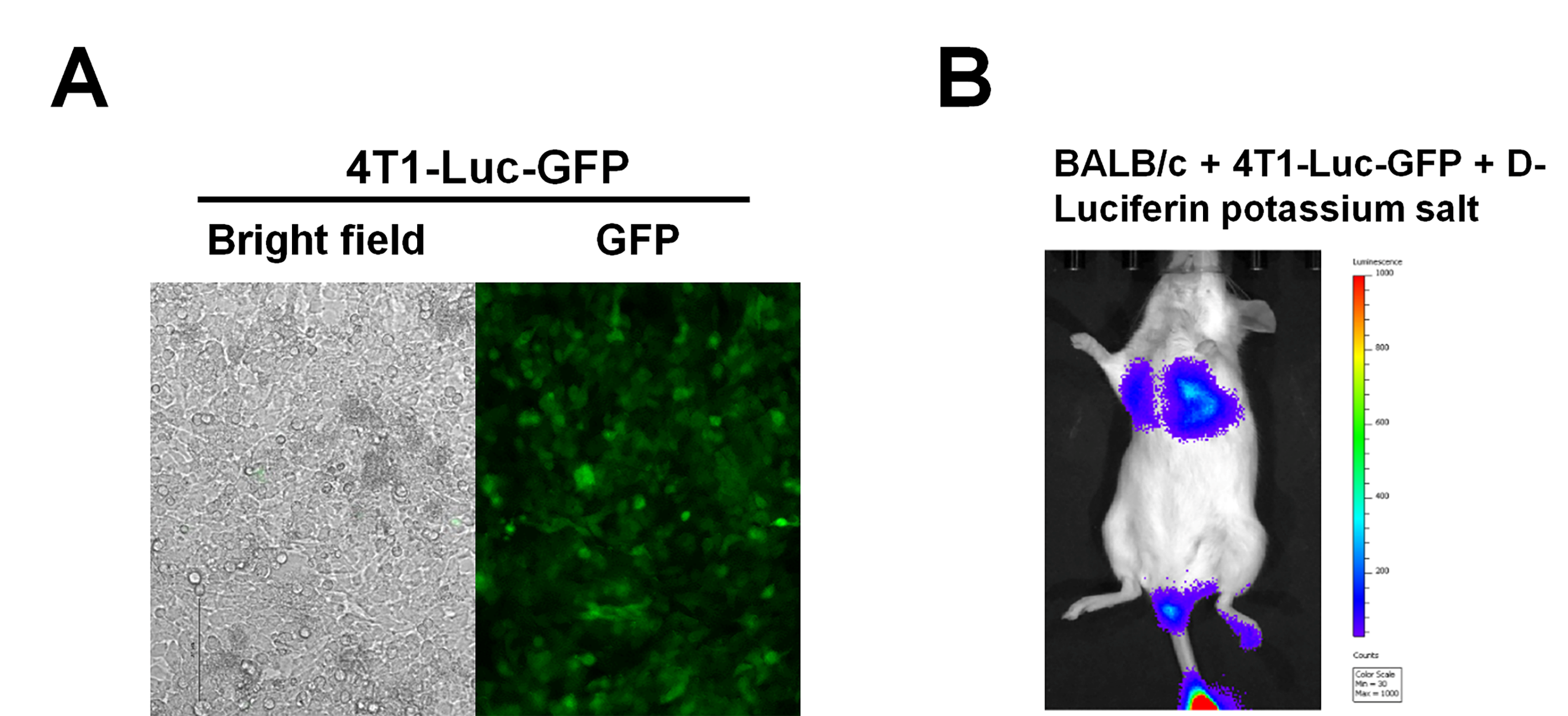
**

**Supplementary Figure 10. The construction of 4T1-Luc-GFP cells.** (**A**) 4T1-Luc-GFP cells were a luciferase-GFP-expressing cell line derived from parent line 4T1 by infection with lentivirus containing the luciferase-GFP-expression cassette and selected by puromycin. The expression of GFP in the cells was detected by a florescent microscope (200×, Thermo, EVOS M5000). Scale bar: 125µm. (**B**) 4T1-Luc-GFP cells (1×10^6^ cells) were injected intravenously into wild-type BALB/c mice, and 7 days later, subjected to intraperitoneal injection of D-Luciferin potassium salt (3 mg/200 mL/mouse) which was dissolved in D-PBS and imaged using an IVIS Lumina XR machine.

**

**

**Supplementary Figure 11.** **M1-20 treatment altered migration-related gene expression levels of metastatic cancers in lung.** (**A**-**B**) Total RNA (**A**) and total proteins (**B**) were prepared with lung tissue on Day 8 after the **M1-20** treatment. Total RNA samples from the three groups were extracted with Trizol reagent. The mRNA levels of E cad and slug were examined by RT-PCR. Relative mRNA levels were normalized to GAPDH. n = 3 for each group, ***P* ≤ 0.01, ****P* ≤ 0.001, *****P* ≤ 0.0001, two-tailed unpaired Student’s t-test. Lung tissues were lysed with RIPA lysis and extraction buffer to obtain whole-cell extracts. The protein levels of E cad and slug were measured by Western blotting.

**
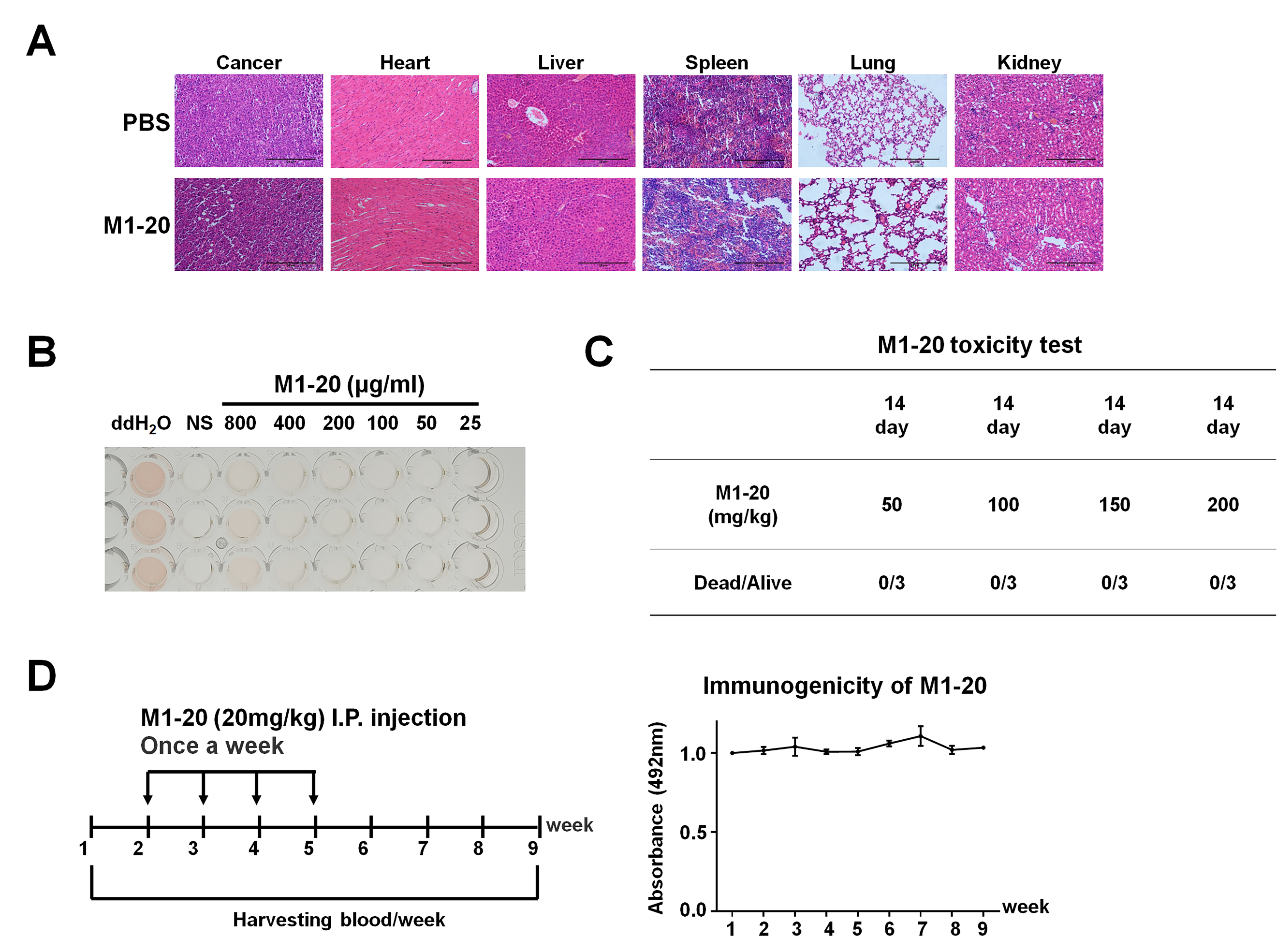
**

**Supplementary Figure 12.** **M1-20 was well tolerated *in vivo*.** (**A**) Representative H&E staining images of different organ slices from mice treated with **M1-20**. Wild-type BALB/c mice were intraperitoneally injected with PBS or **M1-20** (20 mg/kg) once every two days for three weeks. The different organs of the animals were harvested and subjected to tissue segmentation and H&E staining. Photos were taken with an inverted microscope (Olympus IX73). Scale bar: 20 μm. (**B**) Hemolysis analysis of **M1-20**. 10% (v/v) suspension (100 µl per sample) of BALB/c mouse erythrocytes in normal saline (NS, 0.9%) were treated with different concentrations of **M1-20** (25, 50, 100, 200, 400, 800 µg/ml) for 3 h at 37℃. Sterile double distilled water or NS (100 µl) were set as positive or negative controls, respectively. After centrifugation at 10000g for 10 min at 4 ℃, 100 µl supernatant was moved into a 96-well plate and the absorbance was measured at 540 nm using a microplate reader. The hemolytic rate (IR) in response to **M1-20** at different doses was calculated according to the equation: IR % = [(ODsample - ODnegative) / (ODpositive - ODnegative)] × 100%. The percentage of IR versus the concentration of **M1-20** was plotted. The test tubes were taken at the end of the experiment. (**C**) Toxicity test of **M1-20**. ICR/JCL mice (6 weeks old) were randomized into four groups (three mice per group) and injected intraperitoneally with different doses of **M1-20** (50, 100, 150, and 200 mg/kg body weight). The mice were continuously monitored for diet and activity for 14 days. (**D**) Immunogenicity analysis of **M1-20**. ICR/JCL mice (female, 6 weeks, n=3) were injected intraperitoneally with **M1-20** (20 mg/kg) once a week for 4 weeks (Week 2 to Week 5). Blood samples (Week 1 to Week 9) were collected from mice at different time points and the absorbance (492 nm) of the anti-M1-20 antibody in serum (1:1000 dilution) was measured by ELISA. HRP-conjugated anti-mouse IgG was used as ELISA secondary antibody.

**Supplementary Methods**

**Construction of plasmids**

The cDNA of EGFP was PCR amplified from pEGFP-C2 plasmid (Addgene) with primers containing EcoRⅠ and BamHI restriction sites (sense primers: 5’-CCG GAA TCC ATG GTG AGC AAG GGC GAG GA-3’ and antisense primers: 5’-CGG GAT CCT TAC TTG TAC AGC TCG T-3’) and ligated into the pcDNA-3.1 vector (Thermo Fisher Scientific, USA) to obtain the plasmid pcDNA3.1-GFP (pGFP). The cDNA of FOXM1(689-748aa) was PCR amplified from pCMV-FOXM1 (pFOXM1) plasmid [1] with primers containing XbalI and EcoRⅠ restriction sites (sense primers: 5’-GCT CTA GAA TGC CGG AGC CAC AGG TTT CT-3’ and antisense primers: 5’-GCG AAT TCC TGT AGC TCA GGA ATA AAC T-3’) and ligated into the pGFP vector to obtain the plasmid pGFP-FOXM1_689-748_. The cDNA of FOXM1(337-748aa) was PCR amplified from pCMV-FOXM1 plasmid with primers containing XbalI and EcoRⅠ restriction sites (sense primers: 5’-TGC TCT AGA ATG ACC ATC AAA ACC GAA CTC-3’ and antisense primers: 5’-GCG AAT TCC TAC TGT AGC TCA GGA ATA AAC T-3’) and ligated into the pcDNA3.1 vector to obtain the plasmid pFOXM1_337-748_. The cDNA of FOXM1(580-748aa) was PCR amplified from pCMV-FOXM1 plasmid with primers containing XbalI and EcoRⅠ restriction sites (sense primers: 5’- TGC TCT AGA ATG GGA CCT TTT AAG ACA CCC A-3’ and antisense primers: 5’-GCG AAT TCC TAC TGT AGC TCA GGA ATA AAC T-3’) and ligated into the pcDNA3.1 vector to obtain the plasmid pFOXM1_580-748_. The cDNA of FOXM1(1-688aa) was PCR amplified from pCMV-FOXM1 plasmid with primers containing XbalI and EcoRⅠ restriction sites (sense primers: 5’- TGC TCT AGA ATG AAA ACT AGC CCC CGT-3’ and antisense primers: 5’-GCG AAT TCC TAG GAG CCT GGC TTG GGG ACG T-3’) and ligated into the pcDNA3.1 vector to obtain the plasmid pFOXM1_1-688_. The cDNA of FOXM1(689-748aa) was PCR amplified from pCMV-FOXM1 plasmid with primers containing BamHI and XholI restriction sites (sense primers: 5’-CGC GGA TCC ATG TCC CCG GAG CCA CAG GTT-3’ and antisense primers: 5’-CCG CTC GAG CTA CTG TAG CTC AGG AAT-3’) and ligated into the pGEX-4T2 vector to obtain the plasmid pGEX-4T2-FOXM1_689-748_. The cDNA of FOXM1(1-138aa) was PCR amplified from pCMV-FOXM1 plasmid with primers containing BamHI and XholI restriction sites (sense primers: 5’-CGC GGA TCC ATG AAA ACT AGC CCC-3’ and antisense primers: 5’-CCG CTC GAG CTA CAG GGT CAC TTC TGT-3’) and ligated into the pGEX-4T2 vector to obtain the plasmid pGEX-4T2-FOXM1_1-138_. The cDNA of EGFP was PCR amplified from pEGFP-C2 plasmid (Addgene, USA) with primers containing EcoRI restriction sites (sense primers: 5’-GGC TGA TAT CGG ATC CGA ATT CAT GGT GAG CAA GGG CGA G -3’ and antisense primers: 5’-CTT GTC GAC GGA GCT CGA ATT CTT ACT TGT ACA GCT CGT C-3’) and homologous recombination ligated into the pET-32a (Novagen, Germany) vector to obtain the plasmid pET-32a-GFP. The cDNA of FOXM1(1-138aa) was PCR amplified from pCMV-FOXM1 plasmid with primers containing BamHI restriction sites (sense primers: 5’-GGC CAT GGC TGA TAT CGG ATC CAT GAA AAC TAG CCC CC-3’ and antisense primers: 5’-GAC GGA GCT CGA ATT CGG ATC CCA GGG TCA CTT CTG TC-3’) and homologous recombination ligated into the pET-32a-GFP (We cloned it earlier) vector to obtain the plasmid pET-32a-GFP-M1(1-138). The cDNA of Luc was PCR amplified from pGL3-Basic Vector plasmid (Promega, USA) with primers containing EcoRI and BamHI restriction sites (sense primers: 5’-GTG AGG ATC TAT TTC CGG TGA ATT CAT GGA AGA TGC CAA AAA CAT-3’ and antisense primers: 5’-GAG GGA GAG GGG CGG GAT CCT TAC ACG GCG ATC TTG CCG C-3’) and homologous recombination ligated into the pLVX-EF1α-IRES-EGFP vector (SiDanSai, China) to obtain the plasmid pLVX-EF1α-Luc-IRES-EGFP. p6×FOXM1Binding-Luc, pPLK1pro(-1.4kb)-Luc, and pCDC25Bpro(-1.8kb)-Luc reporter plasmids were described previously [2, 3].

**Expression and purification of recombinant proteins**

Expression and purification of recombinant protein were performed as previously described [3]. Briefly, the Rosetta/DE3 *E. Coli* cells were transformed with a certain plasmid and cultured at 37 °C in LB medium until the optical density (OD600) reached 0.8, then 1 mM IPTG was added to induce the expression of target protein cultured for another 12 h at 25-28°C. GST, GST-FOXM1_1-138,_ and GST-FOXM1_689-748_ recombinant proteins were purified according to the instructions of Glutathione Sepharose^TM^ 4B (GE Healthcare, USA). GFP-FOXM1_1-138_ recombinant proteins were purified according to the instructions of Ni-Sepharose^TM^ 6 Fast Flow (GE Healthcare, USA).

**Cell culture**

HEK293T, MDA-MB-231, MCF7, ZR-75-30, MCF10A, Hela, U2OS, A549, and 4T1 cells were obtained from ATCC (Manassas, USA). All cell lines were cultured in Dulbecco’s Modified Eagle Media (DMEM, Gibco) containing 10% fetal bovine serum (Gibco) and 1% penicillin-streptomycin (Invitrogen). All cells were grown in a 5% CO2-humidified atmosphere at 37 °C.

**Luciferase reporter assay**

HEK293T cells were seeded on 12-well plates and transfected with expression plasmids (0.3 µg), certain luciferase reporter vectors (0.9 µg), and pRL-CMV plasmid (20 ng) using EZ trans (LIFE iLAB BIO, Shanghai, China). After transfection for 48h, cells were lysed in 100 µl 1× lysis buffer and the luciferase enzyme activities were measured with the Dual-Luciferase Reporter Assay System (Promega, USA) following the manufacturer's instructions. Luciferase assay-based peptide screening was performed as that the indicated peptides were added to the culture when replaced with fresh medium after 12 hours of transfection and incubated for an additional 24 h to measure dual Luciferase enzyme activity.

**Solid-phase synthesis of peptides**

All reagents and solvents, including dimethylformamide (DMF), dichloromethane (DCM), diethyl ether, methanol, ethanol, and acetonitrile, were purchased from Sinopharm Chemical Reagent Co., Ltd. Triisopropylsilane (TIS), 1,2-Ethanedithiol (EDT), and trifluoroacetic acid (TFA) were obtained from Macklin (Shanghai). Fmoc-AA-OH, Fmoc-D-AA-OH, and 2-Chlorotrityl Chloride Resin were purchased from CS Bio (Shanghai) Ltd. Peptides were generated in a solid phase synthesizer (CS Bio) with 2-Chlorotrityl Chloride resin as solid phase support followed instrument operation program. The synthesized peptides were purified by reverse phase chromatography (AKTA Purifier, GE, USA). The molecular weight of the peptides was identified by Matrix-Assisted Laser Desorption/Ionization Time of Flight Mass Spectrometry (UltrafleXtreme, BURKER, Germany) and the purity of the peptides was determined by Reversed Phase High-Performance Liquid Chromatography (LC-2010, RAINBOW, China).

**Structural modeling with Rosetta FlexPepDock**

The N-terminal structure of FOXM1 (PDB code 6OSW) was obtained from the Protein Data Bank database (PDB, <https://www.rcsb.org>). Rosetta FlexPepDock [4] was used to generate the structural model of the peptide and FOXM1. The best model from 10,000 docking simulations was selected using energy minimization based on docking free energy (dG) calculation with Rosetta InterfaceAnalyzer [5] and PyMOL was used to visualize the binding interface between peptides and proteins. The negative value of the selected dG was used to represent the binding affinity between the peptide and the target protein. Rosetta Flex ddG [6] was used for single-point mutation with alanine scanning to estimate the effect of mutations. The free energy change (ΔΔG) was calculated as the average score difference between the 10 top-scoring mutant and WT structures. Stability calculations were repeated at least 10 times for each mutant, and the mean ΔΔG was used for making stability predictions (ΔΔG≥1, binding energy decreases; 1＞ΔΔG＞-1, binding energy no changes; ΔΔG≤-1, binding energy increases).

**Microscale thermophoresis assay**

GFP-labeled FOXM1_1-138_ protein was mixed with different concentrations of peptide (ranging from 0.030 to 1000 μM) in PBS containing 0.05% Tween-20. Fused silica capillaries (NanoTemper, Germany) were loaded for about 4-6 µl of each sample. Measurements were performed at 25℃ by a Monolith NT.115 instrument (NanoTemper) at a constant LED power of 40% and the MST power of the medium. The data were analyzed by MO. Affinity Analysis v2.3 NT software (NanoTemper) to determine interaction parameters. Signal-to-noise ratios above 10 were considered statistically significant. Data point binding curves from three independent MST measurements are shown, which indicated the fraction of peptide-bound GFP-protein (ΔNormal/Amplitude) at varied ligand concentrations and curves indicated the calculated fits. Error bars showed the Standard Error of three independent measurements.

**Pull-down and Co-immunoprecipitation (Co-IP) Assays**

For pull-down experiments, His-tagged recombinant proteins bound to Ni-Sepharose^TM^ 6 Fast Flow (GE Healthcare, USA) were incubated with cell lysates (500 µg) at 4°C overnight. The pull-downs of His-tagged proteins were analyzed by Western blotting. Biotin-labeled peptides were added to 20 μl Streptavidin Agarose Resin (GE Healthcare, USA) and incubated with GST-tagged recombinant proteins or cell lysates, at 4°C for 4 hours. The resins were washed three times with pre-cooled PBS and subjected to Western blotting.

For Co-IP experiments, Flag magnetic beads (Bimake, USA) were washed three times with TBS, and then 500 µg cell lysates were incubated with 20 µl Flag magnetic beads overnight at 4 °C. The beads were washed three times by TBST (TBS containing 1% Tween-20) and prepared for Western blotting.

**RNA Sequencing**

MDA-MB-231 cells were seeded into 6-cm dishes and treated with **M1-20** (10 µm) or **M1-20mut** (10 µm) for 24 h. The cells were collected into TRIzol reagent (Invitrogen, USA) and sent to Majorbio (Shanghai, China) for RNA extraction and sequencing. The library preparation and Illumina HiSeq platform sequencing were subsequently carried out by the company. Differential expression of genes (DEGs) and gene set enrichment analysis (GSEA) was analyzed using the R package DEGseq [7] on Studio R (version 3.6.1) and GSEA_4.1.0. Gene sets with | log2(fold change) | > 1 and *p*. adjust < 0.1 were considered statistically significant. Pathways with normalized enrichment score |NES| > 1 and *p*-value < 0.05 were considered significantly enriched.

**Protein extraction and Western blotting**

The cells were washed with ice-cold 1×PBS and lysed on ice with IP lysis buffer containing 20 mM Tris/HCl pH 7.6, 150 mM NaCl, 1% NP-40, 0.1 mM EDTA, and protease inhibitors. Tumor tissue samples were homogenized, grinded, and lysed on ice with RIPA buffer (50 mM Tris-HCl pH 8.0, 150 mM NaCl, 2 mM EDTA pH 8.0, 10 mM NaF, 20% glycerol, 1% NP-40 plus protease inhibitors). After centrifugation, the protein concentration of lysates was quantified using a BCA Protein Assay kit (Thermo Fisher Scientific, USA). To extract cytoplasmic and nuclear proteins, cell pellets with 6 cm plates (> 90% confluence) were harvested in CE buffer (10 mM HEPES, 60 mM KCl, 1 mM EDTA, 0.075% (v/v) NP-40, 1mM DTT and 1 mM PMSF, adjusted to pH 7.6), incubated on ice for 5 min and centrifuged for the separation of cytoplasmic proteins. Then wash the nuclei with CE buffer, and resuspended the nuclear pellet in NE buffer (20 mM Tris-Cl, 420 mM NaCl, 1.5 mM MgCl2, 0.2 mM EDTA, 1 mM PMSF, and 25% (v/v) glycerol, adjusted to pH 8.0), adjust the salt concentration to 400 mM using 5 M NaCl, add pellet volume of NE buffer, and vortex to resuspend the pellet, incubate the extract on ice for 10 minutes to obtain the nuclear proteins.

For Western blotting, samples were denatured with protein loading buffer containing beta-mercaptoethanol and heated to 95 ℃ for 10 min. Then, protein lysates were separated by SDS-PAGE gel electrophoresis and transferred to 0.22 µm PVDF membranes (Merck Millipore, USA), followed by Western blotting with certain antibodies. Antibody information is available in Supplementary Table S1. Reactive proteins were detected with an ECL system (GE, USA) by Kodak 4000 MM Imaging System (Kodak). The bands of Western blotting were quantified by ImageJ software.

**RNA isolation and real-time quantitative PCR (RT-PCR)**

TRIzol reagent (Invitrogen, USA) was used to extract the total RNA of cells according to the manufacturer's instructions. cDNA was synthesized from total RNA (2 μg) by reverse transcription (Thermo Fisher Scientific, USA) according to the instructions provided by the manufacturer. RT-PCR was performed using SYBR QPCR Master Mix (Vazyme) with certain sense (S) and antisense (AS) primers, and realplex2 qPCR system (Eppendorf, Germany). The information on RT-PCR primer pairs was presented in Table S2.

**Confocal imaging**

HEK293T cells were plated into glass bottom dishes at approximately 20% confluency. After 12 h, cells were transfected with pRFP-FOXM1 and pGFP-FOXM1_689-748_ and cultured for 48 h. Cells were washed twice with PBS, and fixed with 4% paraformaldehyde at room temperature for 30 min. After washing three times with PBS, the cells were treated with an antifade mounting medium with DAPI and subsequently imaged with fluorescence confocal microscopy (Olympus FluoView FV1200).

**Lentivirus construction and infection**

FOXM1-overexpressing cell line (MCF7-OE or Hela-OE) was established according to the procedure described previously [8]. pLVX-IRES-Puro-Flag-FOXM1 was cotransfected into 293T cells with two packaging plasmids psPAX2 (Addgene #12259, USA), and pMD2.G (Addgene #12259, USA) by polyethyleneimine (PEI) transfection reagent (pLv/psPAX2/pMD2G = 12 μg/9 μg/6 μg) to produce lentiviruses. Hela or MCF7 cells were infected with the lentivirus and positive cells were screened by puromycin (1 μg/mL).

**Cell proliferation assay**

Cells (4×10^3^/well) were seeded into 96-well plates in triplicate, and 12 h later cultured with different concentrations of **M1-20** at 37℃. After another 36 h, cell viability was measured using Cell Counting Kit-8 Solution (CCK8, Bimake, USA) at 450 nm wavelength (OD450). The inhibition ratio and IC50 were calculated using GraphPad Prism 9 software (GraphPad Prism Software Inc., San Diego, CA, USA).

EdU cell proliferation staining was performed using an EdU kit (BeyoClick™ EdU Cell Proliferation Kit with Alexa Fluor 488, Beyotime, China) following the manufacturer’s protocol. Briefly, MDA-MB-231 cells (2 × 10^4^ cells/well) were seeded in 24-well plates and treated with **M1-20** (10 µm), **M1-20mut** (10 µm) or non-treated for 24 h. Next, cells were incubated with EdU for 2 h, fixed with 4% paraformaldehyde for 15 min, and permeated with 0.3% Triton X-100 in PBS for another 15 min. The cells were incubated with 100µl Click Reaction Mixture for 30 min at room temperature without light, then stained nucleus with Hoechst 33342 for 20 min and photographed with a fluorescence microscope.

**Cell cycle analysis**

MDA-MB-231 cells (2×10^5^) were seeded in 6-cm dishes for 12 h and treated with **M1-20** (10 µm), **M1-20mut** (10 µm), or non-treated. After incubation for 24 h, the cells were harvested by trypsinization, washed with ice-cold PBS twice, and fixed with 70% ethanol at 4°C for 30 min. Cells were resuspended and stained with propidium iodide (PI) without light for 30 min at room temperature according to the manufacturer's protocols (Beyotime, China). The cells were ﬁltered through 70-μm cell strainers and analyzed for DNA content on a ﬂow cytometer (Beckman Coulter).

**Wound-healing assay**

MDA-MB-231 cells were seeded in a 12-well plate at 2.8 × 10^5^ cells per well. A 200 µl pipette tip was used to scratch a line when cells were in full confluence. The cells were rinsed twice with PBS and photographed at 0 h. Cells were then treated with **M1-20** (10 µm) or **M1-20mut** (10 µm) and cell migration was recorded at different times after wound formation. According to the imaging results, the cell migration area was calculated by ImageJ software, and the cell migration rate was calculated by GraphPad software.

**Apoptosis assay**

A One Step TUNEL Apoptosis Assay Kit (Beyotime, Shanghai, China) was used to detect apoptotic cells according to manufacturers’ instructions. MDA-MB-231 cells (2 × 10^4^ cells/well) were seeded in 12-well plates and treated with **M1-20** (10 µm), **M1-20mut** (10 µm) or non-treated for 24 h. Next, cells were fixed with 4% paraformaldehyde for 30 min, washed twice with PBS, and permeated with 0.3% Triton X-100 in PBS for another 15 min. Then, cells were incubated with 50µl TUNEL detection mixture for 1h at room temperature without light. Cell nuclei were stained with DAPI. Fluorescence images were observed with fluorescence microscopy and the percentage of TUNEL-positive cells was calculated with Image J software.

An Annexin V-FITC Apoptosis Detection Kit (Beyotime, Shanghai, China) was used to detect apoptotic cells according to manufacturers’ instructions. MDA-MB-231 cells were treated with **M1-20** (10 µm), **M1-20mut** (10 µm), or non-treated for 24 h. Cells were harvested and stained with Annexin V-FITC and PI for 20 min without light. The cells were analyzed with a ﬂow cytometer (Beckman Coulter).

**Electrophoretic mobility shift assays (EMSAs)**

The FAM-labeled and unlabeled DNA probes were synthesized by Sangon (Shanghai) Co., Ltd, China, based on the following sequence, DNA probe: forward strand 5’-FAM-TTT GTT TAT TTG TTT GTT TAT TTG-3’(Hot) or 5’-TTT GTT TAT TTG TTT GTT TAT TTG-3’(Cold), and reverse strand 5’-CAA ATA AAC AAA CAA ATA AAC AAA-3’. Proteins (2 µm) were mixed with the FAM-labeled dsDNA probe (50 nm) in EMSA binding buffer (20 mM Tris-Cl, 50 mM KCl, 10% glycerol, 0.5 mM EDTA, 0.2 mM DTT, pH 7.6) for 30 min on ice. The unlabeled cold probe DNA (5 µM) or **M1-20** of increased dosage (0.5, 1, and 2 µM) used for a competitive experiment or interaction experiment were added to the reactions. The reactions were performed in 4% native polyacrylamide gel electrophoresis in 0.5×TBE buffer and imaged with Kodak 4000 MM Imaging System (Kodak) (EX: 465 nm, EM: 535 nm for FAM).

**The anti-cancer effects of M1-20 *in vivo***

All animal care and experiments were performed by the guidelines approved by the Laboratory Animal Center of Hunan, China (Protocol No. SYXK [Xiang] 2018-0006). Healthy ICR/JCL mice (6-week-old), BALB/c mice (female, 4-week-old), and BALB/c nude mice (female, 4-week-old) were purchased from Hunan Slac Laboratory Animal Company (Changsha, China). To study the inhibitory ability of **M1-20** on the tumorigenicity of cancer cells *in vivo*, BALB/c nude mice (n=7) were subcutaneously injected with MDA-MB-231 cells (1×10^7^ cells/mouse) suspended in 150 µl PBS into the right axilla. When the tumor reached about 20 mm^3^, the mice were randomly divided into the **M1-20** group (n=4) and **M1-20mut** group (n=3). Peptides (4 mg/kg) were injected directly into tumors (orthotopic injection) every other day for 29 days (14 times total). Meantime, the subcutaneous tumor volume (V) and body weight was measured with a vernier caliper and a balance twice a week, and the tumor volume (V) was calculated according to the following formula: V = 1/2 × length × width^2^. At the end of the experiment, all nude mice were sacrificed by ethyl ether and photographed. Tumors were extracted, weighed, and photographed. To further investigate the influence of **M1-20** on cancers in wild-type mice, 4T1 cells (1 × 10^6^ cells/injection) in 0.2 ml PBS were subcutaneously implanted into the left and right flank of BALB/c mice. One week later, mice were randomly divided into three groups (three mice/group), followed by intraperitoneal (I.P.) injection of PBS (n=6), **M1-20** (20 mg/kg, n=6) or **M1-20mut** (20 mg/kg, n=6) every other day for 3 weeks. At the end of the experiment, all the mice were sacrificed by ethyl ether, and the tumor was extracted, weighed, and photographed. Tumors were prepared for RNA isolation, Western blotting, IHC, and tissues were used for HE staining. The detailed procedure was described in Supplementary Materials and Methods. The 4T1-Luc-GFP cells (5 ×10^4^ cells/mouse) were intravenously administered into BALB/c mice to induce metastasis. Three days later, mice were randomly divided into three groups, followed by intraperitoneal injection of PBS (n=17), **M1-20** (10 mg/kg, n=7), or **M1-20** (20 mg/kg, n=17) every two days until the end of the experiments. At different time points after **M1-20** treatment (Day 7 and Day 11), *in vivo* imaging of three randomly selected mice was performed by intraperitoneal injection of D-Luciferin potassium salt (3 mg/200 μL/mouse) with IVIS Lumina XR machine (Caliper, USA). Different organs of representative mice were harvested on Day 8 for the lumina imaging, and lungs were used for HE staining, RNA isolation, and Western blotting. When the mice lost mobility and had difficulty eating, the mice were considered near death and terminated according to animal welfare. The survival statistic curve was obtained by the Mantel-Cox estimator with the log-rank test.

**Immunohistochemistry**

The tumor tissues separated from mice were washed by PBS, ﬁxed with 4% paraformaldehyde, dehydrated, and embedded in paraﬃn. IHC was performed on 5 µm-thick tumor slices. Then, after deparaffinization, rehydration, antigen retrieval, and endogenous peroxidase blockage following as manufacturer’s instructions, slides were incubated separately with primary antibodies: PCNA (1:100) and Caspase 3 (1:100), overnight at 4 °C. The slides were rinsed with a nonspecific binding antibody with PBS and then incubated with HRP-conjugated secondary antibody (ZSGB-Bio, China) at room temperature for 30 min, washed three times with PBS again. DAB (ZSGB-Bio, China) was used for staining. The nucleus was stained with hematoxylin. The slices pictures were taken at ×200 magniﬁcation using an inverted microscope (Olympus IX73).

**Histological and morphological analyses**

Mice organs and tissues were separated, fixed in 4% paraformaldehyde overnight at 4℃, dehydrated, embedded in paraﬃn, and frozen at −80 °C for 3 h. The tissues were serial 5 µm thick sections prepared and stained with Hematoxylin and Eosin Staining Kit (HE, Beyotime, China) to assess the histology and morphology of organs and tissues in the experimental animals. The stained specimens were visualized using an inverted microscope (Olympus IX73).

**Hemolysis Assays**

Fresh mouse blood was centrifuged at 2500 rpm for 5 min to collect red blood cells. A 10% suspension of erythrocytes (v/v) was suspended in normal saline (NS, 0.9%). The suspension (100 µl/sample) was treated with diﬀerent concentrations of **M1-20** (0, 25, 50, 100, 200, 400, 800 µg/ml) for 3 h at 37 °C, while positive and negative control samples were respectively prepared by adding an equal volume of sterile double distilled water (ddH_2_O) and NS into the suspension. After 3 h, the samples were centrifuged at 10, 000g for 10min at 4 °C, and 100 µl of supernatants were added to a 96-well plate. The absorbance of each well at 540 nm was read on a microplate reader (Bio Tek) for calculating hemolysis percentages. Hemolysis rate % = [(OD sample - OD negative) / (OD positive - OD negative)] × 100%.

**Toxicity test and immunogenicity assays of M1-20**

For the acute toxicity test, mice were randomized into four groups (three mice per group) and injected with different doses of **M1-20** (50 mg/kg, 100 mg/kg, 150 mg/kg, or 200 mg/kg) by intraperitoneal injection. The control group was injected intraperitoneally with PBS. The mice were continuously monitored for diet and activity for 14 days. Immunogenicity of **M1-20** was performed by ELISA in mice. ICR/JCL mice (n = 3) were injected intraperitoneally with **M1-20** (20 mg/kg) once a week for 4 weeks (Week 2 to Week 5). At different time points (Week 1 to Week 9), blood samples were collected from mice. The **M1-20** peptide was dissolved in the coating buffer and coated at a concentration of 1 μg/well for 12 h at 4°C. ELISA plates were washed twice with PBS and blocked with 3% bovine serum albumin for 2 h. After washing with PBS, the collected serum of mice from weeks 1 to 9 was diluted 1:1000 to measure the change of anti-**M1-20** antibody concentration in serum. HRP-conjugated anti-mouse IgG was used as ELISA secondary antibody. The absorbance was measured at 492 nm.

**Statistical analysis**

Data analysis and visualization were performed using Microsoft Excel and GraphPad Prism 9 (GraphPad Software Inc.). The analysis was tested by unpaired t-test between 2 groups, and by one-way ANOVA or two-way ANOVA with multiple comparisons in multiple groups. Significant differences between the control and experimental groups were assessed with **P* < 0.05; ***P* < 0.01; *** *P* < 0.001; **** *P* < 0.0001.

**References**

1. Xie ZQ, Tan GX, Ding MA, Dong DF, Chen TH, Meng XX, et al. Foxm1 transcription factor is required for maintenance of pluripotency of P19 embryonal carcinoma cells. Nucleic Acids Res. 2010;38(22):8027-38.

2. Xiang Q, Tan G, Jiang X, Wu K, Tan W, Tan Y. Suppression of FOXM1 Transcriptional Activities via a Single-Stranded DNA Aptamer Generated by SELEX. Sci Rep. 2017;7:45377.

3. Zhang Z, Bu H, Yu J, Chen Y, Pei C, Yu L, et al. The cell-penetrating FOXM1 N-terminus (M1-138) demonstrates potent inhibitory effects on cancer cells by targeting FOXM1 and FOXM1-interacting factor SMAD3. Theranostics. 2019;9(10):2882-96.

4. Raveh B, London N, Schueler-Furman O. Sub-angstrom modeling of complexes between flexible peptides and globular proteins. Proteins. 2010;78(9):2029-40.

5. Stranges PB, Kuhlman B. A comparison of successful and failed protein interface designs highlights the challenges of designing buried hydrogen bonds. Protein Sci. 2013;22(1):74-82.

6. Barlow KA, S OC, Thompson S, Suresh P, Lucas JE, Heinonen M, et al. Flex ddG: Rosetta Ensemble-Based Estimation of Changes in Protein-Protein Binding Affinity upon Mutation. J Phys Chem B. 2018;122(21):5389-99.

7. Wang L, Feng Z, Wang X, Wang X, Zhang X. DEGseq: an R package for identifying differentially expressed genes from RNA-seq data. Bioinformatics. 2010;26(1):136-8.

8. Yang C, Chen H, Tan G, Gao W, Cheng L, Jiang X, et al. FOXM1 promotes the epithelial to mesenchymal transition by stimulating the transcription of Slug in human breast cancer. Cancer Lett. 2013;340(1):104-12.

**Supplementary Table S1.**

| **REAGENT or RESOURCE** | **SOURCE** | **USAGE** | **IDENTIFIER** |
| --- | --- | --- | --- |
| **Antibodies and Purification Beads** | | | |
| Rabbit monoclonal anti-FOXM1 (clone D3F2B) | Cell Signaling Technology | 1:2000 (WB) | Cat# 20459S |
| Rabbit polyclonal anti-β-Actin (D8) | Bioworld Technology | 1:10000 (WB) | Cat# AP0731 |
| Rabbit monoclonal anti-DYKDDDDK Tag (clone D6W5B) | Cell Signaling Technology | 1:1000 (WB) | Cat# 14793 |
| Rabbit polyclonal anti-FLAG Tag | Beyotime | 1:2000 (WB) | Cat# AF0036 |
| Mouse Monoclonal anti-His Tag | Beyotime | 1:2000 (WB) | Cat# AF5060 |
| HRP-labeled Streptavidin | Beyotime | 1:5000 (WB) | Cat# A0303 |
| Rabbit polyclonal anti-GFP | Beyotime | 1:2000 (WB) | Cat# AG279 |
| Mouse monoclonal anti-GST Tag | Sangon Biotech | 1:2000 (WB) | Cat# D190101 |
| Rabbit monoclonal anti-Lamin B1 | Beyotime | 1:1000 (WB) | Cat# AF1408 |
| Rabbit polyclonal anti-β-Tubulin | Beyotime | 1:1000 (WB) | Cat# AF1216 |
| Rabbit polyclonal anti-PLK1 | Proteintech | 1:1000 (WB) | Cat# 10305-1-AP |
| Rabbit monoclonal anti-PLK1 | Beyotime | 1:2000 (WB) | Cat# AG2902 |
| Rabbit monoclonal anti-CyclinB1 | Beyotime | 1:2000 (WB) | Cat# AF1606 |
| Rabbit monoclonal anti-CyclinD1 | Santa Cruz | 1:1000 (WB) | Cat# SC450 |
| Mouse polyclonal anti-Cdc25B | Abcam | 1:2000 (WB) | Cat# ab167347 |
| Rabbit polyclonal anti-PCNA | Sangon Biotech | 1:2000 (WB)  1:100 (IHC) | Cat# D220014 |
| Rabbit polyclonal anti-Bax | Sangon Biotech | 1:2000 (WB) | Cat# D220073 |
| Rabbit Monoclonal anti-Caspase 3 (active) | Beyotime | 1:1000 (WB)  1:100 (IHC) | Cat# AF1150 |
| Rabbit polyclonal anti-E-Cadherin | Cell Signaling Technology | 1:1000 (WB) | Cat# 3195S |
| Rabbit monoclonal anti- Vimentin | Beyotime | 1:2000 (WB) | Cat# AF1975 |
| Rabbit polyclonal anti-ALDH1 | Sangon Biotech | 1:2000 (WB) | Cat# D120058 |
| Rabbit polyclonal anti-CD44 | Sangon Biotech | 1:2000 (WB) | Cat# D122619 |
| Rabbit monoclonal anti-Slug | Cell Signaling Technology | 1:1000 (WB) | Cat# C19G7 |
| Mouse monoclonal anti-LIN9(C-10) | Santa Cruz | 1:500 (WB) | Cat# sc-398234 |
| Rabbit polyclonal anti-CBP | Abclonal | 1:2000 (WB) | Cat# A1334 |
| Horseradish peroxidase-conjugated goat anti-rabbit IgG(H+L) | Beyotime | 1:2000 (WB) | Cat# A0208 |
| Horseradish peroxidase-conjugated goat anti-mouse IgG(H+L) | Beyotime | 1:2000 (WB) | Cat# A0216 |
| Anti-Flag Magnetic Beads | Bimake | 20 μL (Each hole) | Cat# B26102 |
| Glutathione Sepharose beads | GE Healthcare | 20 μL (Each hole) | Cat# 17-0756-01-10mL |
| Ni-Sepharose^TM^ 6 Fast Flow | GE Healthcare | 20 μL (Each hole) | Cat# 17-5318-06-5mL |
| Streptavidin Agarose Resin | GE Healthcare | 20 μL (Each hole) | Cat# 17-5113-01-5mL |
| **Bacterial and virus strains** | | | |
| DH5α Competent Cells | Sangon Biotech | Cat# B528413 | |
| Stbl3 Competent Cells | Lab Homemade |  | |
| BL21(DE3) Competent Cells | Sangon Biotech | Cat# B528414 | |
| Lentivirus system | Lab Homemade |  | |
| **Reagents and Kits** | | | |
| Endotoxin-Free Plasmid Mini Extraction Kit | TIANGEN | Cat# DP118-02 | |
| EZ Trans | LIFE iLAB BIO | Cat# AC04L092 | |
| Blood/Cell/Tissue Genomic DNA Extraction Kit | TIANGEN | Cat# DP304-03 | |
| Maxima H Minus First Strand c DNA Synthesis Kit with dsDNase | Thermo Fisher Scientific | Cat# K1682 | |
| In-Fusion Snap Assembly Master Mix | TaKaRa | Cat# 638947 | |
| Dual-Luciferase® Reporter Assay System | Promega | Cat# E1960 | |
| D-Luciferin potassium salt | Beyotime | Cat# ST196 | |
| NHS-Biotin | Sangon Biotech | Cat# C100212-0050 | |
| BeyoClick™ EdU Cell Proliferation Kit with Alexa Fluor 488 | Beyotime | Cat# C0071S | |
| Annexin V-FITC Apoptosis Detection Kit | Beyotime | Cat# C1062S | |
| 2-step plus Poly-HRP Anti Mouse/Rabbit IgG Detection System | ZSGB-Bio | Cat# PV-9000 | |
| DAB solution | ZSGB-Bio | Cat# ZLI-9018 | |
| Hematoxylin and Eosin Staining Kit | Beyotime | Cat# C0105M | |

**Supplementary Table S2.**

| **Gene** | **RT-PCR primers (5ʹ-3ʹ)** | |
| --- | --- | --- |
|  | **Sense primers** | **Antisense primers** |
| GAPDH-human | ACCCAGAAGACTGTGGATGG | TGCTGTAGCCAAATTCGTTG |
| PLK1-human | TTCCCAAGCACATCAACCCCGT | AATGGTTGGGCGGGCAGTGG |
| CDC25B-human | AGTCCTGACCGGAAGATGGA | GATGTTGCTGAACTTGCCCG |
| PCNA-human | GCGTGAACCTCACCAGTATGT | TCTTCGGCCCTTAGTGTAATGAT |
| Ki-67-human | TCCTTTGGTGGGCACCTAAGACCTG | TGATGGTTGAGGTCGTTCCTTGAT |
| E cad-human | CGGGAATGCAGTTGAGGATC | AGGATGGTGTAAGCGATGGC |
| Vim-human | GAGAACTTTGCCGTTGAAGC | GCTTCCTGTAGGTGGCAATC |
| Bax-human | CCCGAGAGGTCTTTTTCCGAG | CCAGCCCATGATGGTTCTGAT |
| Caspase 3-human | CATGGAAGCGAATCAATGGACT | CTGTACCAGACCGAGATGTCA |
| GAPDH-mouse | GATGCTGGTGCTGAGTATGRCG | GTGGTGCAGGATGCATTGCTCTGA |
| PLK1-mouse | CCCGCTGGCGAAAGAAATTC | CATTTGGCGAAGCCTCCTTTA |
| CDC25B-mouse | CACTCTTACACAGACCATGCAC | ACGACAGGCTTCCTACTTGAG |
| PCNA-mouse | TTTGAGGCACGCCTGATCC | GGAGACGTGAGACGAGTCCAT |
| Bax-mouse | CGGCGAATTGGAGATGAACTG | AGCAAAGTAGAAGAGGGCAACC |
| Caspase 3-mouse | TGAAGGGGTCATTTATGGGACA | CCAGTCAGACTCCGGCAGTA |
| E cad-mouse | TCGGAAGACTCCCGATTCAAA | CGGACGAGGAAACTGGTCTC |
| slug-mouse | CAGCGAACTGGACACACACA | ATAGGGCTGTATGCTCCCGAG |
