# Supplementary material for: Development of an interfering peptide M1-20 with potent anti-cancer effects by targeting FOXM1
Source: Cell Death Dis. 2023 Aug 19;14(8):533. doi: 10.1038/s41419-023-06056-9 (PMC10439915; doi:10.1038/s41419-023-06056-9)

Full and uncropped western blot for Figure 1B

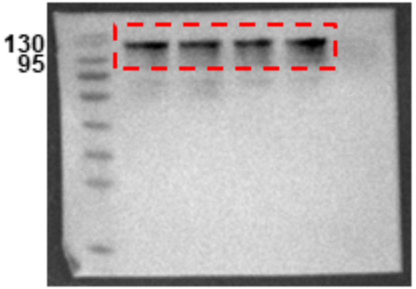

IB: Flag

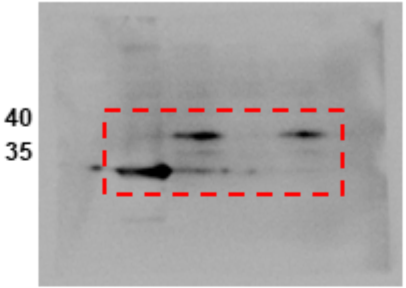

IB: GFP

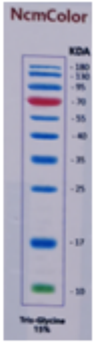

Full and uncropped western blot for Figure 1C

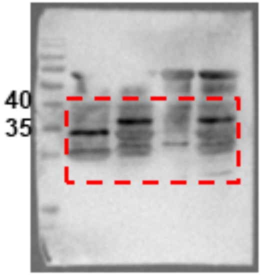

IB: Flag

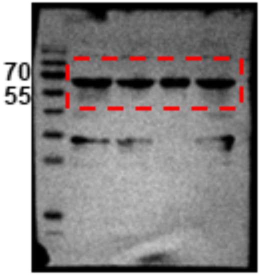

IB: His

Full and uncropped western blot for Figure 2A

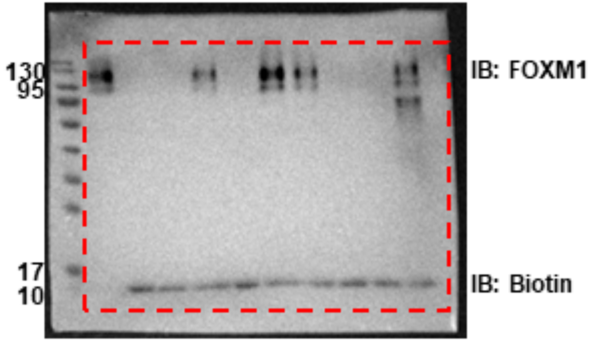

Full and uncropped western blot for Figure 2C

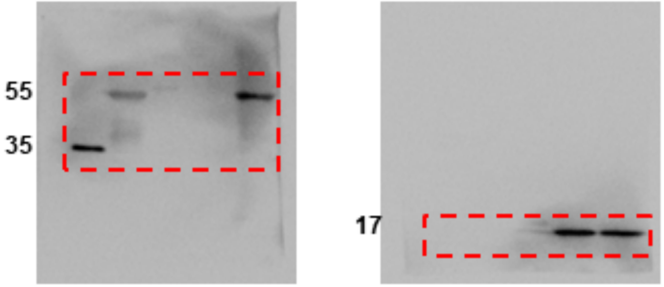

IB: GST

IB: Biotin

Full and uncropped western blot for Figure 2F

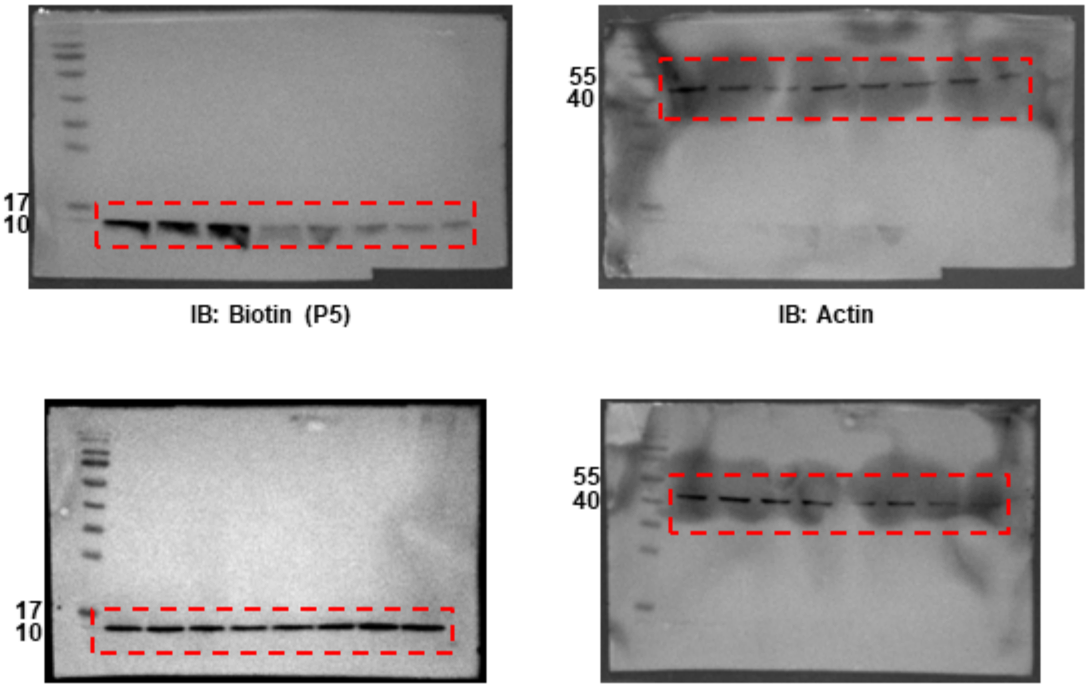

IB: Biotin (P5)

IB: Actin

IB: Biotin (M1-20)

IB: Actin

Full and uncropped western blot for Figure 2H

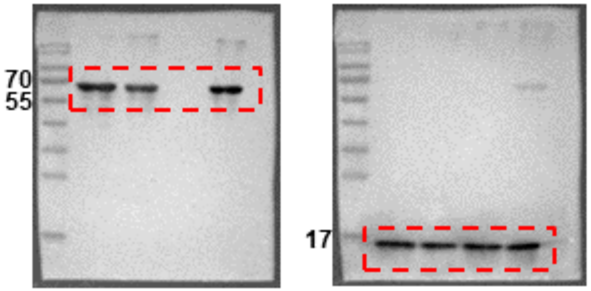

IB: GFP IB: Biotin

Full and uncropped western blot for Figure 4C, G, K

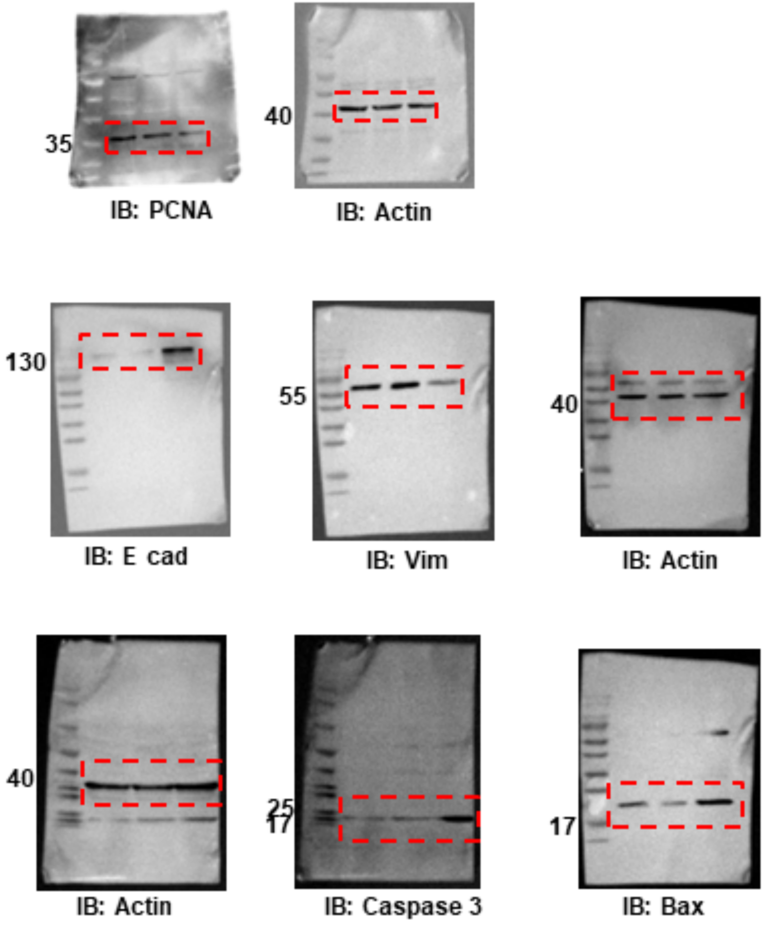

Full and uncropped western blot for Figure 4P

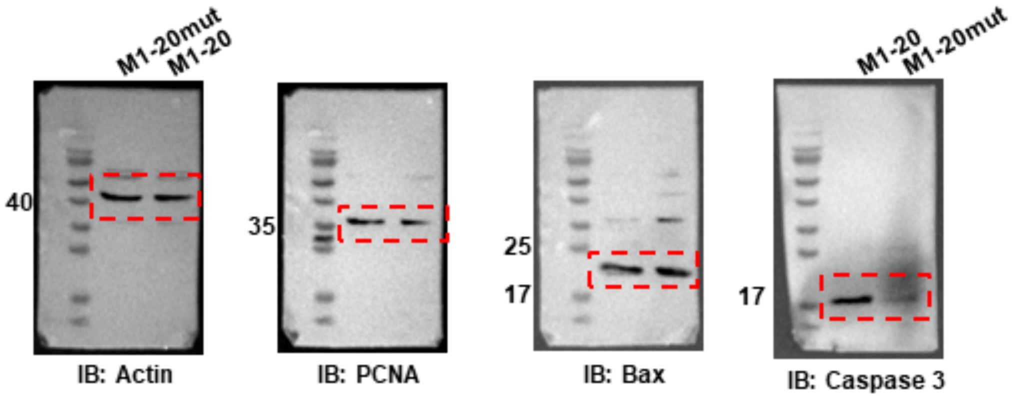

Full and uncropped western blot for Figure 5A

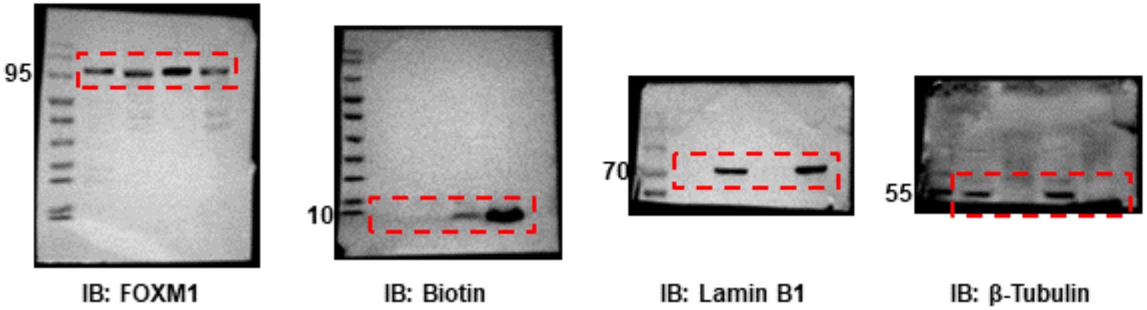

Full and uncropped western blot for Figure 5B

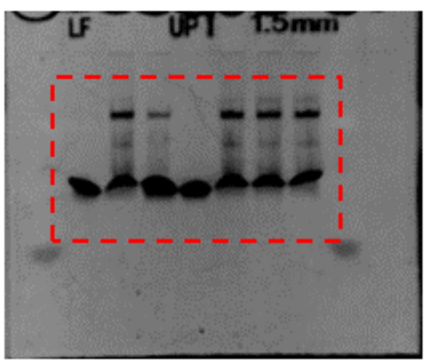

Full and uncropped western blot for Figure 5C

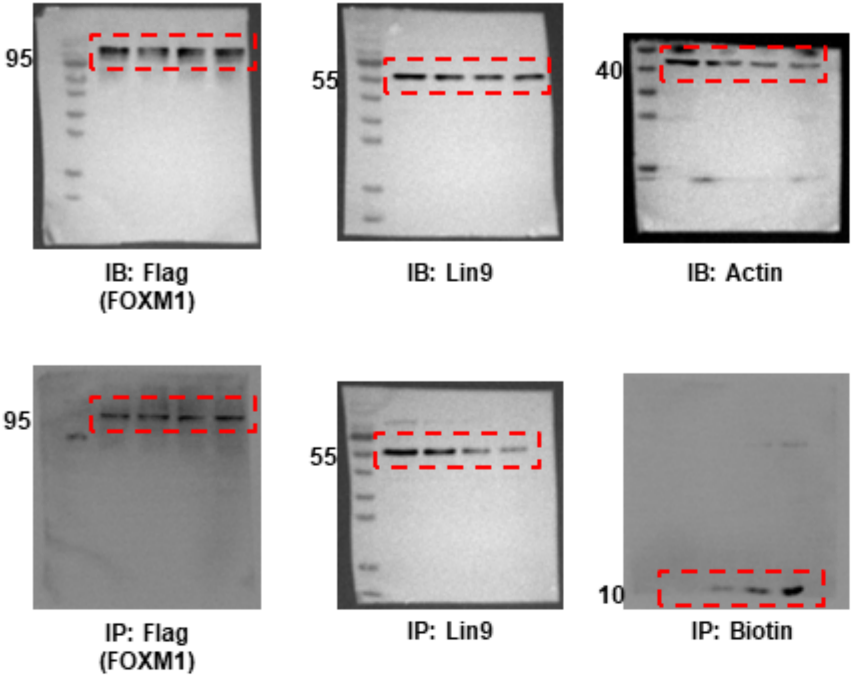

Full and uncropped western blot for Figure 5E

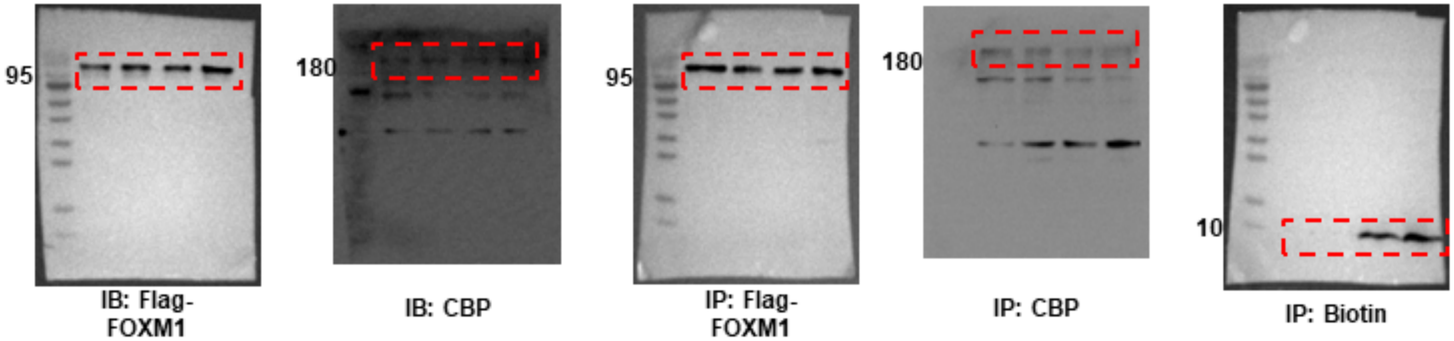

Full and uncropped western blot for Figure S1B

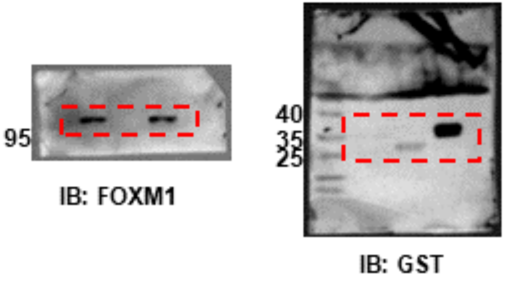

Full and uncropped western blot for Figure S5A

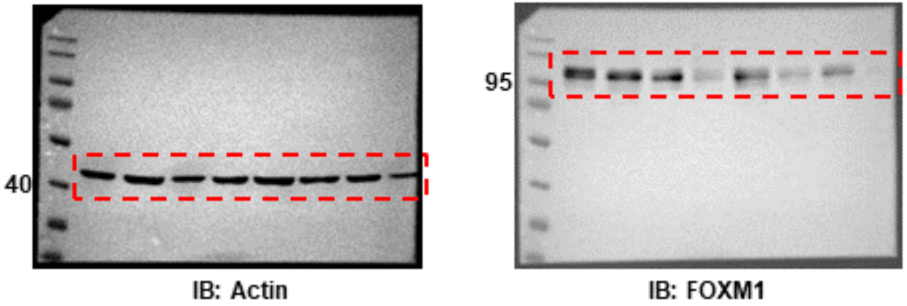

Full and uncropped western blot for Figure S5B-C

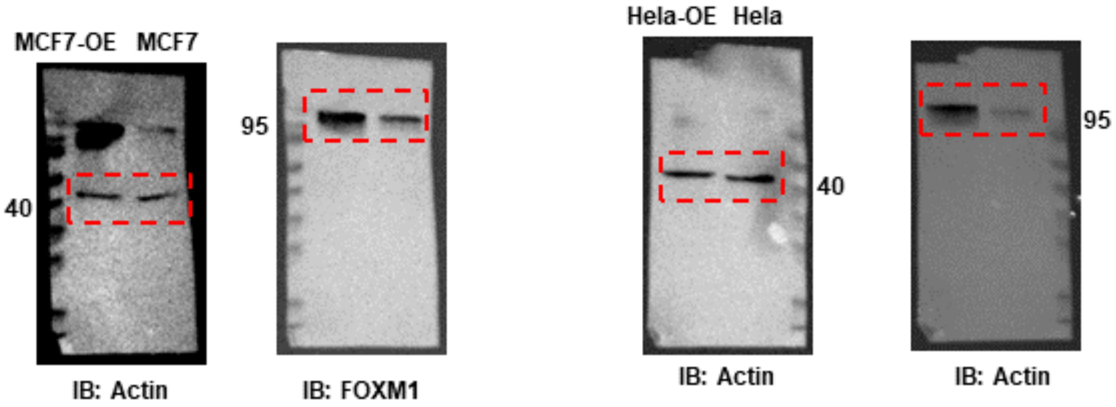

Full and uncropped western blot for Figure S6D

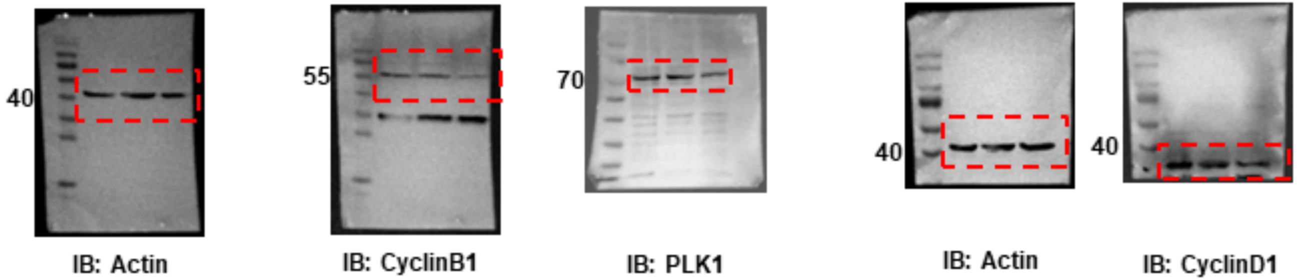

Full and uncropped western blot for Figure S6H

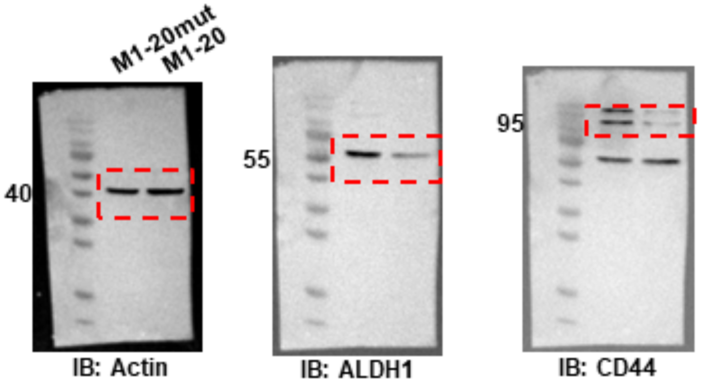

Full and uncropped western blot for Figure S8B

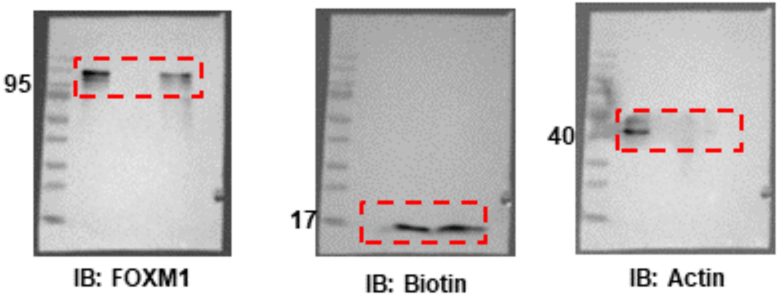

Full and uncropped western blot for Figure S9B

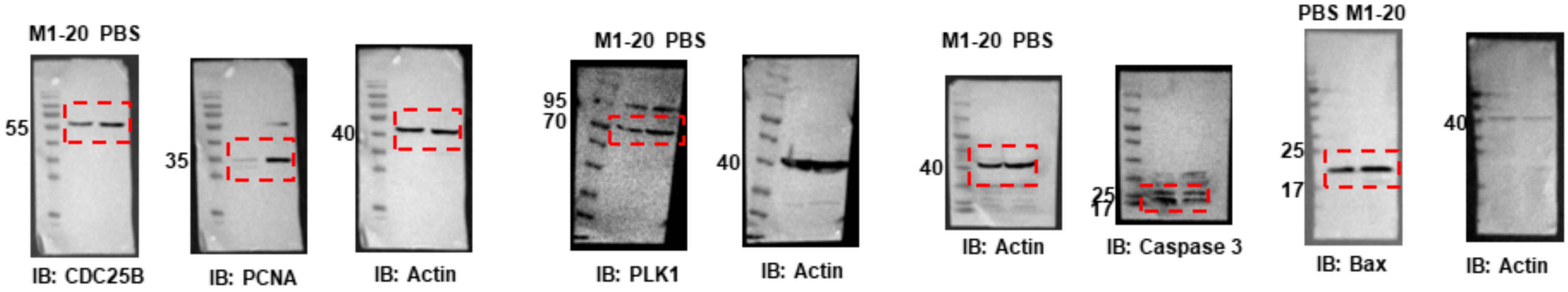

Full and uncropped western blot for Figure S11B

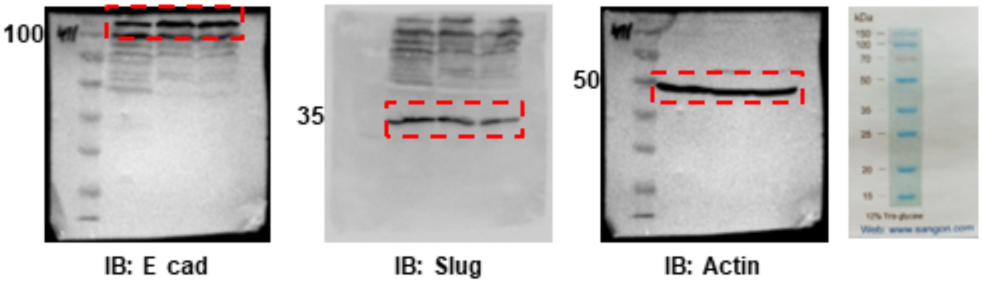

Supplement: Supplementary file 2 — Original WB data [file 41419_2023_6056_MOESM2_ESM.pdf]
